# Visual Coding in Locust Photoreceptors

**DOI:** 10.1371/journal.pone.0002173

**Published:** 2008-05-14

**Authors:** Olivier Faivre, Mikko Juusola

**Affiliations:** 1 Department of Zoology, University of Cambridge, Cambridge, United Kingdom; 2 Department of Biomedical Science, University of Sheffield, Sheffield, United Kingdom; Centre de Recherches su la Cognition Animale - Centre National de la Recherche Scientifique and Université Paul Sabatier, France

## Abstract

Information capture by photoreceptors ultimately limits the quality of visual processing in the brain. Using conventional sharp microelectrodes, we studied how locust photoreceptors encode random (white-noise, WN) and naturalistic (1/*f* stimuli, NS) light patterns *in vivo* and how this coding changes with mean illumination and ambient temperature. We also examined the role of their plasma membrane in shaping voltage responses. We found that brightening or warming increase and accelerate voltage responses, but reduce noise, enabling photoreceptors to encode more information. For WN stimuli, this was accompanied by broadening of the linear frequency range. On the contrary, with NS the signaling took place within a constant bandwidth, possibly revealing a ‘preference’ for inputs with 1/*f* statistics. The faster signaling was caused by acceleration of the elementary phototransduction current - leading to bumps - and their distribution. The membrane linearly translated phototransduction currents into voltage responses without limiting the throughput of these messages. As the bumps reflected fast changes in membrane resistance, the data suggest that their shape is predominantly driven by fast changes in the light-gated conductance. On the other hand, the slower bump latency distribution is likely to represent slower enzymatic intracellular reactions. Furthermore, the Q_10_s of bump duration and latency distribution depended on light intensity. Altogether, this study suggests that biochemical constraints imposed upon signaling change continuously as locust photoreceptors adapt to environmental light and temperature conditions.

## Introduction

Sensory systems face the challenge of reliably encoding the outside world as neural signals in the face of an ever-changing environment. A classical example is the light adaptation of the visual system over a vast range of intensities - the ability to ‘disregard’ redundant mean illumination so that contrast patterns can be encoded within the limited output range of neurons [1]–[3]. Insect compound eyes, which allow stable intracellular recordings from their visual neurons in the presence of intact circuitry and optical structures, make particularly useful models to study light adaptation [4]–[14], providing the opportunity to investigate how a sensory system extracts information from its surroundings, and how this function is optimized to environmental changes [2], [3], [9], [15], [16].

Photoreceptors constitute the sensory surface of insect compound eyes, the retina. In these cells, light patterns are encoded into graded membrane potentials for transmission through the first visual synapse [4], [5], [9], [10], [13]–[15], [17]. This starts a parallel flow of signals that is relayed several times [18], [19] before this neural image reaches the brain. The quality of the neural image at the photoreceptor level is critical to the animal's survival, as any higher-order processing by the brain ultimately relies on this representation of the visual scenery [1], [2].

In insect photoreceptors the absorption of a photon by rhodopsin leads to the initiation of ionic currents, and these currents elicit changes in the membrane potential [12]. The voltage signal is therefore co-processed by the phototransduction cascade and the membrane [11], both having their properties dynamically regulated [7], [8]. The resulting plastic, adaptive ‘gain control’ has evolved to work efficiently, despite several limiting factors. The following constraints are of particular relevance: (1) the *noisiness* of both the light input, such as photon shot-noise and optical blur, and the cellular machinery, such as chemical reaction dynamics and ion channel kinetics; (2) the *vast range of light intensities* to which the animal is exposed, threatening to saturate the small operational voltage range of a photoreceptor; and (3) the *ambient temperature*, which acutely affects the speed of intracellular reactions as most insects are poikilothermal. Our aim in this study is to quantify how locust photoreceptors encode visual information *in vivo* and how this process is affected by these three major parameters: noise, light background (BG) and temperature.

The photoreceptors of the desert locust (*Schistocerca gregaria*) offer several advantages in investigating how a sensory system reliably encodes information in a changing, noisy environment. First, the ecology and behavior of locusts is well-characterized [20]. In their natural habitat in Africa, these animals are active both by day and night. Therefore, locusts not only have to adapt to very different light BGs but they also face large temperature changes, making it biologically meaningful to investigate how these factors impact the way photoreceptors encode contrast signals. Secondly, their relatively large photoreceptors allow stable, long-lasting intracellular recordings [21]–[24]. One can therefore reliably repeat the experiment in the same cell at different temperatures and light BGs. Thus, we can unambiguously distinguish between variability attributable to the changing mean illumination and/or ambient temperature and variability attributable to cell-to-cell differences.

In this study, we quantify the response dynamics of locust photoreceptors to random (white-noise, WN) and naturalistic (1/*f*, NS) contrast stimuli at different light BGs and temperatures. We also investigate how their membrane properties change with these conditions by injecting current waveforms intracellularly. This combined approach allows us to elucidate the respective roles and intricate tuning of the phototransduction cascade and plasma membrane in shaping the voltage responses to light contrasts. We show that the temperature-dependence of different biochemical processes involved varies with light adaptation. Nevertheless, we also find that the locust photoreceptors are able to produce a remarkably invariable neural representation of naturalistic light patterns, irrespective of the prevailing light and temperature conditions. Based on our results, we reason that temporal input patterns continuously tune the interactions between the fast membrane reactions (bump waveform) and the slower intracellular reactions (bump latency distribution), enabling the speed of the voltage output to encode contrast values of the input. By accurately encoding the naturalistic contrast input into the rate of change of voltage responses (and so generating an invariable bandwidth for NS), the locust photoreceptors provide robust neural representations of the natural environment already at the first stage of neural information processing.

## Materials and Methods

### Preparation

Adult female locusts (*Schistocerca gregaria*) were reared in the Department of Zoology in the University of Cambridge. The culture contained 500–1,000 insects per 45×50×50 cm rearing units and was maintained under an 18 h∶6 h light∶dark cycle. The temperature during the light period was 37°C and during the dark period 25°C.

Dissection: the pronotum was carefully removed, the head cut off and its back sealed with beeswax to prevent it from drying. The antennae and mouthparts were delicately removed to avoid muscular saccades. The head was then fixed with beeswax to the open end of a conical holder, mounted on top of a ceramic recording platform. Two openings were cut on the head with a sharp razor edge. The first one, a size of a few ommatidia, was made on the dorsal cornea of the left eye and sealed with Vaseline to prevent the eye from drying. The second, a larger one on the top of the head, was used to implant the indifferent electrode. Despite the dissection, the health of the preparation was excellent, providing with very stable recordings. If it were correctly sealed, the head could survive many hours - when left overnight the preparation was still alive; it responded electrically to light the following day. All the experiments were realized during the mid-afternoon, when the animals were in their ‘day state’ [25]–[27].

### Temperature control

The hollow copper core of the holder was shielded within a ceramic insulator and fitted tightly onto a Peltier element. Heat sink paste was used to enhance heat conduction. Underneath the Peltier element, a large copper rod embedded in ice functioned as a heat sink. The temperature of the head was measured with a thermocouple, mounted in the copper core next to the head. A custom-designed power source, controlled by the feedback from the thermocouple, was used to drive the Peltier element. The room temperature was monitored with a separate thermocouple. Control measurements from the head revealed that its temperature depended linearly on the temperature of the copper holder at a given room temperature. The actual temperature of the head was estimated from a reliable calibration, using the measured temperature values of the thermocouple at the constant room temperature of 19°C (constantly monitored and controlled by air conditioning). All the experiments were realized with an actual temperature of the head ranging from 13 to 25°C. Although the behaviorally relevant temperatures for *Schistocerca gregaria* certainly extend to higher temperatures, stabilizing the preparation temperature with such a differential from the room temperature proved technically difficult. This range of temperature was nevertheless sufficient to accurately estimate Q_10_ values (the rate of change as a consequence of increasing the temperature by 10°C) for various parameters.

### Microelectrodes and cell selection

The microelectrodes were pulled with a horizontal laser puller (P-2000; Sutter Instrument Company) from filamented quartz glass capillaries (Sutter, with an inner and outer diameter of 0.5 and 1.0 mm, respectively). Electrodes were back-filled with 3 M KCl, having resistance between 80 and 180 MΩ in the tissue. Microelectrodes were mounted on a manual micromanipulator (HB3000R; Huxley Bertram) and entered the compound eye through the previously prepared small hole. A blunt reference microelectrode, filled with locust Ringer's containing in mM: 10 TES buffer, 140 NaCl, 10 KCl, 4 CaCl_2_, 4 NaHCO_3_, 6 Na_2_HPO_4_, adjusted to pH 6.8 with NaOH/HCl [28], entered the locust's head through the other opening.

**Figure 1:**
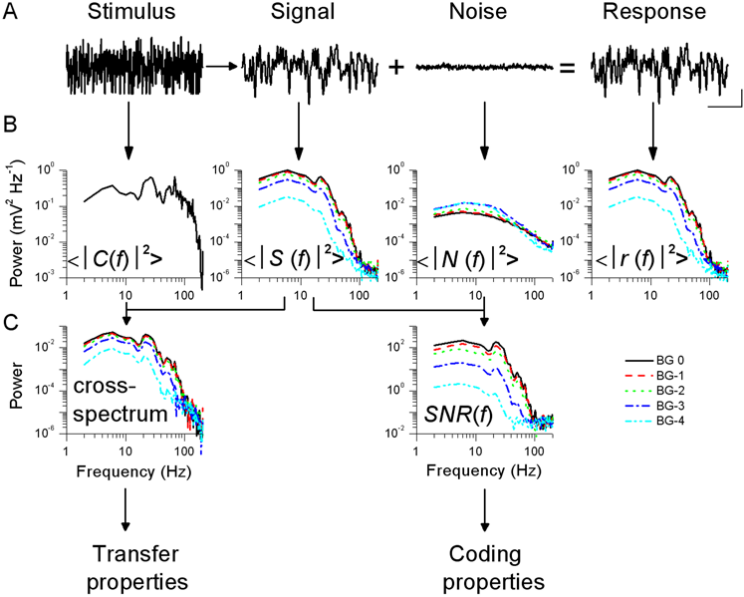
Signal and noise analysis of the voltage responses to a white-noise (WN) light stimulus. A, A pseudorandom light intensity pattern superimposed on a constant light background provided a WN contrast stimulus that was presented 30 times to the cell. The evoked responses are averaged to give the voltage signal and the remaining differences are the noise traces (A, scale bars: 500 ms, 5 mV). B, The corresponding power spectra are calculated for each of the five light BGs. Note that 〈|*S*(*f*)|^2^〉, 〈|*N*(*f*)|^2^〉, and 〈|*r*(*f*)|^2^〉 are displayed using the same scale, in mV^2^ Hz^−1^. 〈|*C*(*f*)|^2^〉 is in c^2^ Hz^−1^. C, These changes can be further quantified by computing the signal-to-noise ratio spectrum, *SNR*(*f*), and the cross-spectrum between the signal and the stimulus. These two spectra are the starting points to quantify the properties of the photoreceptor voltage responses (Figs. 2 and 3), the *SNR*(*f*) being used for the analysis of the coding properties (Fig. 2) and the cross-spectrum for the analysis of the transfer properties (Fig. 3). ‘Power’ on the ordinate scale of the cross-spectrum means here c mV Hz^−1^.

Membrane potentials of green-sensitive R1–R6 photoreceptor cells [29], [30] were recorded with a switched-clamp amplifier SEC-10L (NPI Electronic) operating in the compensated current-clamp mode. A successful photoreceptor penetration was seen as a 60–80 mV drop in the electrode potential followed by vigorous responses to dim pulses. Before the experiments, the cells were allowed to dark-adapt and seal properly. Only data from photoreceptors with saturating impulse responses ≥40 mV and dark resting potential ≤−60 mV were used in the analysis. In this article, we exhibit our findings using two exemplary photoreceptors. Similar results were obtained from other photoreceptors (n = 15) that endured long-lasting recordings. These data are presented in the Supporting Information. A first photoreceptor is used throughout Materials and Methods to illustrate the way data was analyzed (Figs. 1 to [Fig pone-0002173-g002] 3) at a constant temperature (19°C). The second one is used throughout the article (Figs. 4 to [Fig pone-0002173-g005] [Fig pone-0002173-g006] [Fig pone-0002173-g007] [Fig pone-0002173-g008] [Fig pone-0002173-g009] [Fig pone-0002173-g010] [Fig pone-0002173-g011] 12). Because of its exceptional stability, we were able to use this cell in many separate experiments and so to explore how light adaptation occurs over a vast range of background intensities and temperatures (from 17 to 23°C). For these experiments we used both white-noise (WN) and naturalistic stimulation (NS), and were able to further investigate how the membrane properties of the cell varied at each experimental condition. Additionally, we made recordings from many other photoreceptors (>30 of outstanding quality) over a smaller range of experimental conditions. These recordings were consistent with the general framework presented here. Because we believe that intrinsic functional variability between photoreceptors could be an important feature of locust vision (see Discussion), we do not show averaged quantities. Data from these cells is shown as Q_10_ values in Table 1 and detailed further in Table S1.

**Figure 2 pone-0002173-g002:**
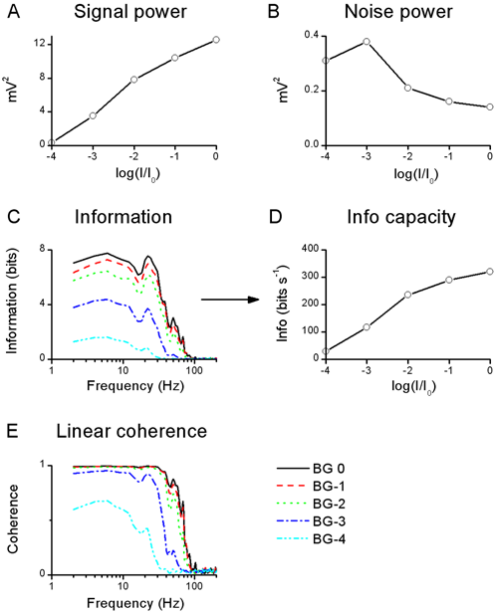
Voltage responses to a WN light stimulus: analysis of the coding properties of the photoreceptor. Analysis of the coding properties of the photoreceptor, based on the *SNR*(*f*) (Fig. 1C, right). A, The total signal power increases ∼40 times from BG-4 to BG0. The variance calculated in the time domain, σ*_S_* ^2^, (not shown) is virtually identical, verifying the calculations. B, The total noise power is reduced ∼2 times from BG-4 to BG0. Here again it is identical to the noise variance calculated in the time domain, σ*_N_* ^2^, (not shown). C, Information in the frequency domain is calculated from *SNR*(*f*) at each frequency as log_2_[*SNR*(*f*)+1]. All the information resides in a frequency range below 100 Hz. This information is integrated to give the information transfer rate (Shannon's formula), D, which increases ∼11 times from BG-4 to BG0. The ratio of the signal and noise variances, *SNR_t_* (not shown) scales well with the information transfer rate. This highlights that the information transfer rate is a measure of the number of the ‘coding states’ used by the cell during a second. These states are the different voltage levels confined within the used voltage range (which is ∼ signal as σ*_S_* ^2^>>σ*_N_* ^2^) and separated one from another by the ‘resolution’ of the system (noise). From information transfer rate estimates we define three relevant backgrounds: BG-3, named as ‘dim’ (∼100 bits/s); BG-2 as ‘mid’ (∼200 bits/s) and BG0 as ‘bright’ (∼300 bit.s^−1^). E, Linear coherence, γ*_lin_*, is calculated from *SNR*(*f*). At dim BGs the stimulus is itself noisy (attributable to the photon shot-noise), and so is the cell's behavior. At bright BGs the cell's response (assuming linearity, see Materials and Methods) is remarkably noise-free (γ*_lin_*>99% at BG0) up to ∼30 Hz.

**Figure 3:**
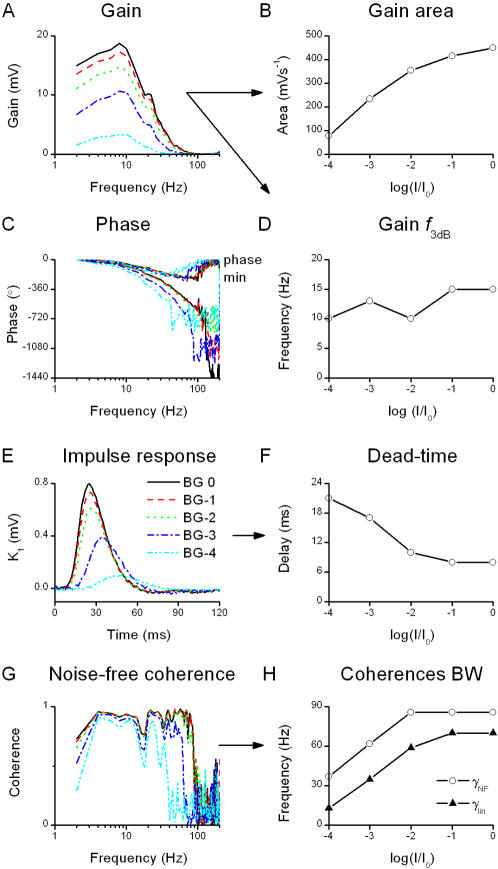
Voltage responses to a WN light stimulus: analysis of the transfer properties of the photoreceptor. Analysis of the transfer properties of the photoreceptor, based on the cross-spectrum between the stimulus and the signal (Fig. 1C, left). A, Gain is the norm of the frequency response (see Materials and Methods). It displays the range and extent of stimulus frequencies the cell amplify linearly. B, Areas (integrals) under gain curves at different BGs, and D, corresponding 3 dB cut-off frequencies. The amplification increases with the light BGs whereas the cut-off frequency remains virtually unchanged. C, Phase of the frequency response and the minimum phase, calculated from the gain curves, exhibits a phase-lag. E, Impulse response *K_1_* is calculated from the frequency response function (real parts seen as gain, A, and phase, C). It approximates the linear filtering properties of the system. Brightening increases its area, scaling closely with the gain power (not shown), and reduces its onset-delay, F, as well as its time-to-peak (the delay between onset and peak is virtually constant ∼20 ms). The dead-times estimated from the phase-shift observed in C (not shown) and from the impulse response (F) behave very similarly, vindicating the analysis. G, Noise-free coherence, γ*_NF_*, indicates the frequency range where a photoreceptor, if operating linearly, would reproduce exactly the same response at each stimulus presentation. γ*_NF_* departs from unity at certain frequencies, reflecting selective nonlinearities, which enhance particular features of the stimulus. The bandwidths of the coherences, H, are defined as the frequency beyond which γ<0.5. The bandwidths increase with brightening BGs, reflecting the photoreceptor's ability to follow the stimulus on a shorter time-scale (γ*_lin_*). This increased precision takes place in a frequency range where the photoreceptor encodes linearly the WN stimulus (γ*_NF_*>γ*_lin_* at each BG).

**Figure 4:**
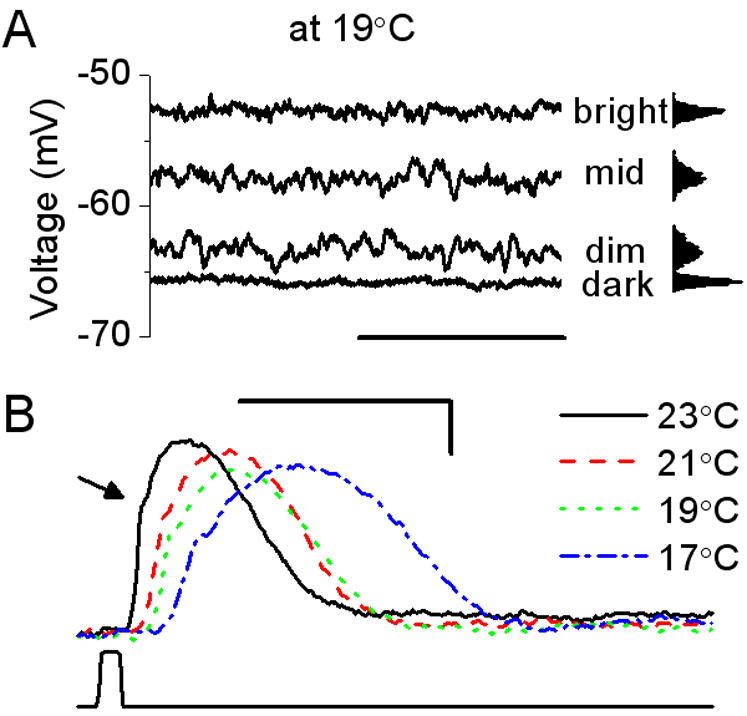
Light background and temperature are critical parameters for the visual coding in locust photoreceptors. A, Light-induced depolarization, at 19°C, is clearly seen in 1 s-long recordings of the membrane potential of a photoreceptor adapted to different light conditions – to darkness and to three different light BGs. Brightening reduces voltage noise, as seen from the corresponding probability distributions (right; scale bar: 500 ms). B, Voltage responses of a dark-adapted photoreceptor to a 10 ms-long light pulse of saturating intensity at 17, 19, 21, and 23°C (scale bars: 100 ms, 10 mV) show that warming accelerates voltage responses to light but has little impact on their amplitude (∼40 mV). The mean potentials have been set to the same value for clarity. The arrow indicates a fast depolarizing transient [29], similar to the ones reported in *Calliphora* [76] and *Drosophila* [7] photoreceptors.

**Figure 5 pone-0002173-g005:**
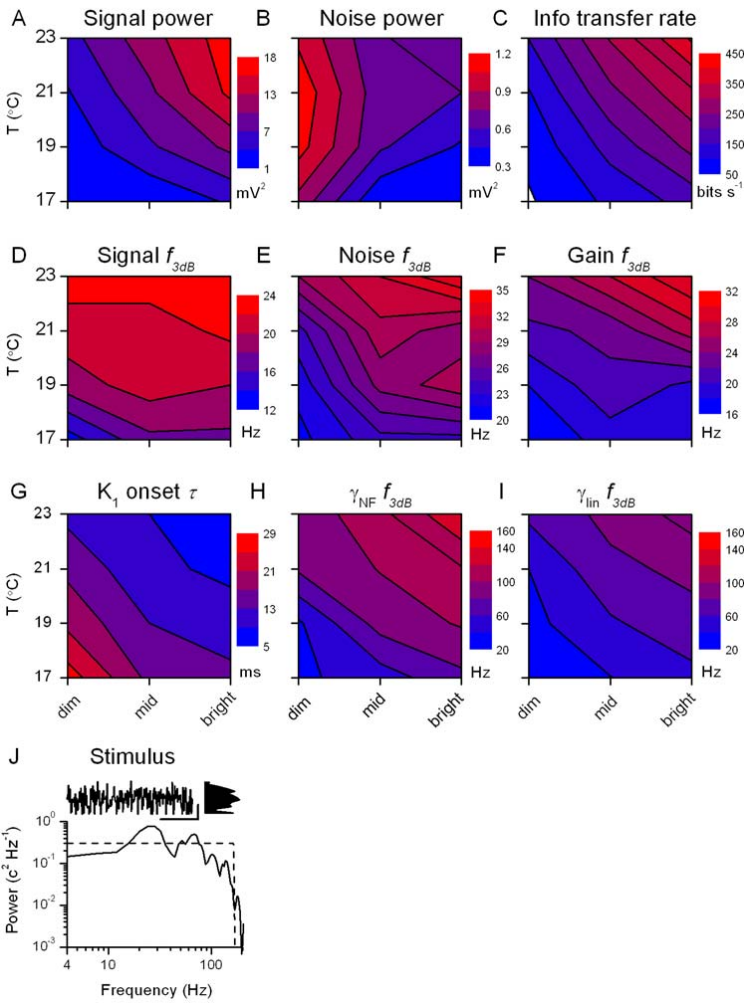
Analysis of the voltage responses to a light WN stimulus at different BGs and temperatures. Changes in signal, A, and noise, B, power with brightening and warming lead to an increase in information transfer rate, C. Warming increases 3 dB cut-off frequencies of the signal, D, noise, E, and gain function, F. Dead-time in the voltage response, as seen with the onset time of the impulse response, G, is also reduced with both warming and brightening. H, Cut-off frequency of the noise-free coherence, γ*_NF_*, i.e. the frequency beyond which γ*_NF_*<0.5, and I, cut-off frequency of the linear coherence, γ*_lin_*, are presented using the same scale, highlighting that for every experimental condition γ*_NF_*>γ*_lin_*. J, WN stimulus can be characterized by its temporal pattern (scale bars: 300 ms, 1 contrast unit), by its probability distribution and by its power spectrum.

**Figure 6 pone-0002173-g006:**
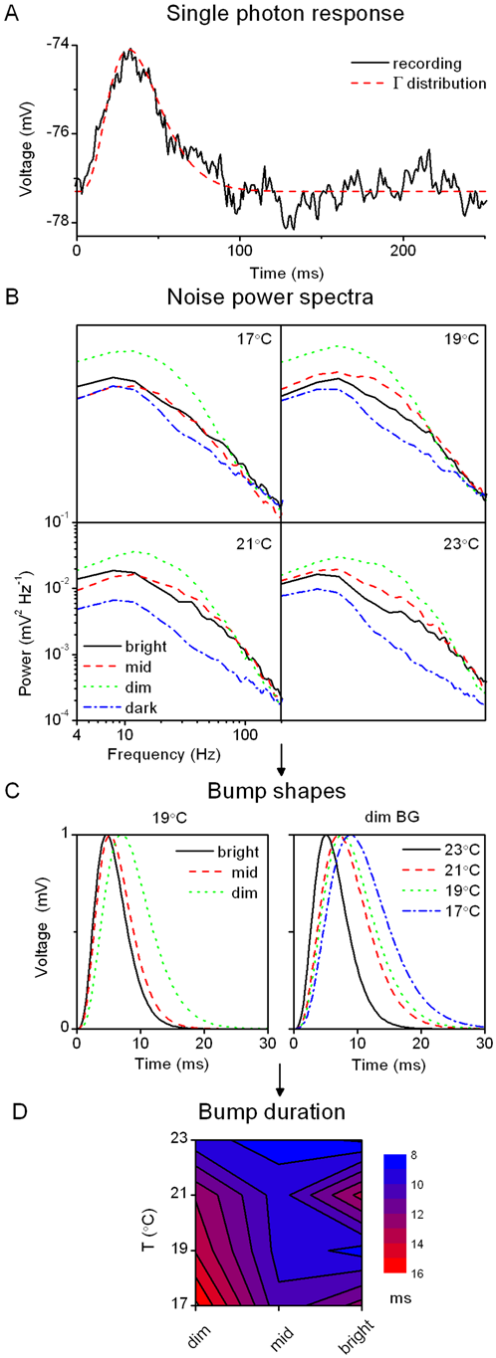
Bump noise analysis of the voltage responses to a WN light stimulus. A, Single photon response recorded in a dark-adapted photoreceptor at 26°C and super-imposed Γ-distribution (n = 4, τ = 8 ms). An initial estimate of these parameters is necessary to guide the fitting algorithm. These first-guess parameters can be estimated by calculating the power spectrum of a single bump and fitting a Lorentzian to obtained curve (see Materials and Methods). For this bump the parameters estimated in the frequency space gave n = 4, τ = 9 ms. The Γ-distribution accurately describes the bump shape. The mean residual of the fit is 0.085 mV^2^ (estimated between 0 and 90 ms, i.e. where the bump is actually happening), smaller than the fluctuations of membrane potential in bump-free zone (variance ∼0.1 mV^2^). B, At a given temperature we estimate the noise spectra of the voltage responses at the three adapting BGs and in darkness. The dark noise is virtually the same over the temperature range; it is subtracted from the total noise at each BG to give the light-induced noise power spectra. By fitting a single Lorentzian to these spectra we obtain parameters for the bump waveform (see Materials and Methods). C, Normalized bump shapes for different BGs at 19°C and for different temperatures at the dim BG illustrate that both brightening and warming accelerate the bumps. D, This is further quantified by estimating the bump durations (or time-to-peak; not shown as it displays identical behavior).

**Figure 7 pone-0002173-g007:**
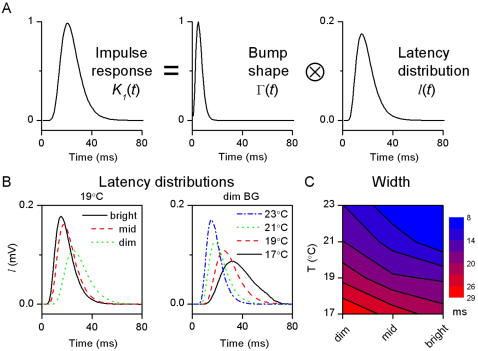
Bump latency distributions. A, Latency distribution is calculated by deconvolving the bump shape from the corresponding impulse response (see Materials and Methods) at each experimental condition. B, Estimated latency distributions are shown for the same conditions as in Figure 6. As their time-to-peak decreases with brightening or warming, bumps appear sooner. C, The bumps are also more precise (synchronized), as it can be seen in the decrease of the width of the (normalized) latency distributions.

**Figure 8 pone-0002173-g008:**
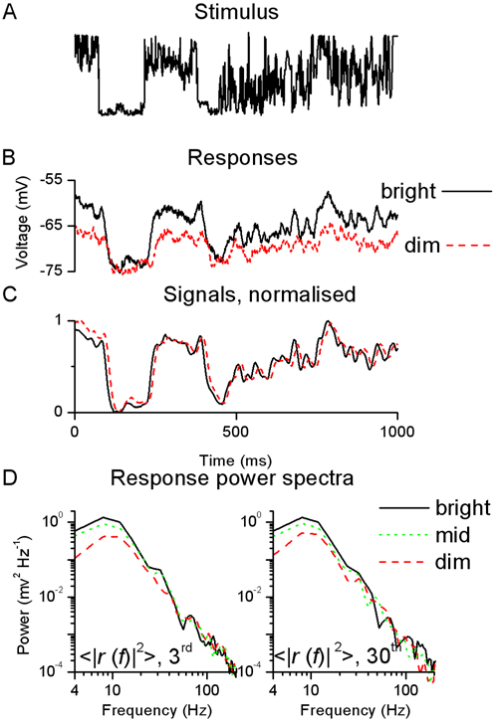
Voltage responses to a NS light pattern at 19°C. A, In separate experiments, a NS light pattern is repeatedly presented to a photoreceptor as dim and bright intensity variations. B, The superimposed traces show the corresponding voltage responses to the 20^th^ stimulus presentations. For the bright NS the cell dedicates a larger voltage range for encoding the stimulus. C, Averaging over the 100 individual traces gives the corresponding signals, normalized to exhibit the differences in their timing. With the dim NS the voltage output of the photoreceptor follows the light input with a delay greater than the one with the bright NS by ∼1 ms. Nevertheless, the voltage responses can follow the same stimulus pattern, suggesting that the photoreceptor is utilizing the same frequency range at different BGs. This is confirmed by the analysis of the responses power spectra at different points during the repeated stimulation (3^rd^ and 30^th^ traces), D. Whilst the amplification is higher for bright NS, as was already apparent in A, the range of frequencies encoded is virtually the same. This is quantified by calculating the 3 dB cut-off frequency, *f*_3 dB_, which equals to 14 Hz in all cases. Comparing the spectra of the responses to the 3^rd^ (left) and 30^th^ (right) stimulation shows no additional adaptive trend, suggesting that the system adapts rapidly (after the 1^st^ stimulation) to a relatively invariable coding state (see Text S1 and Fig. S2). The signal power spectra (not shown) look very similar to the power spectra of the responses, as expected from the high signal-to-noise ratio (Fig. 9).

**Figure 9 pone-0002173-g009:**
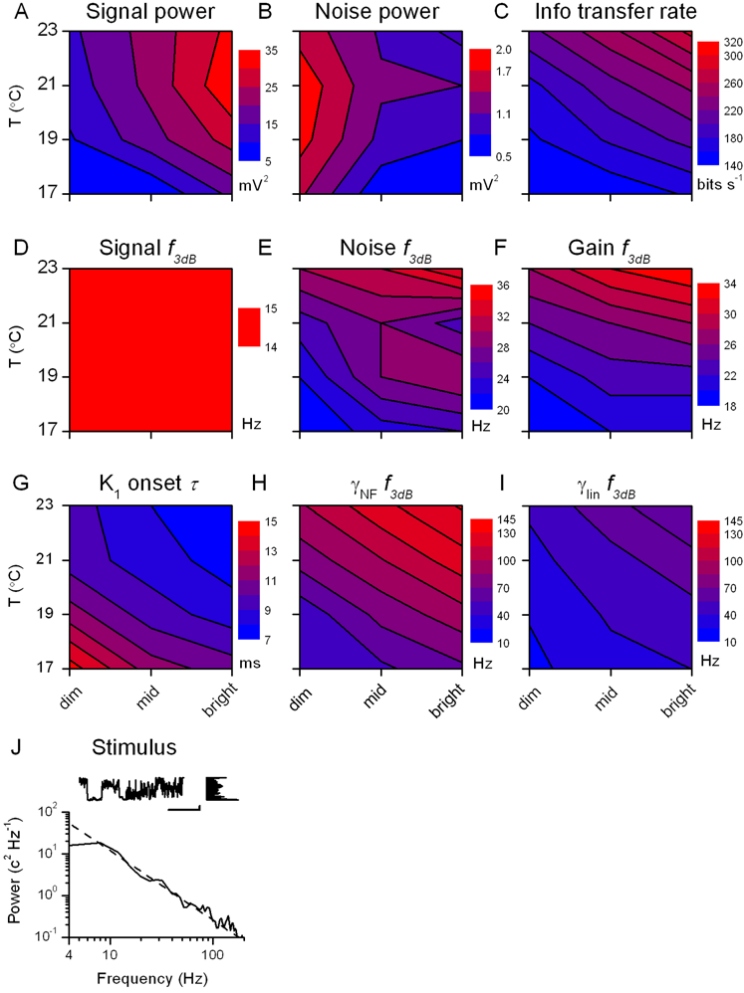
Analysis of the voltage responses to a light NS stimulus at different BGs and temperatures. Responses to a NS light stimulus change with warming and brightening. Behaviors of signal power, A, noise power, B and information transfer rate, C, as estimated with the triple extrapolation method, resemble those of the WN experiment (Fig. 5). D, signal 3 dB cut-off frequency remains virtually unchanged over all the BG-temperature conditions, differing dramatically from the WN experiment, whereas, the cut-off frequencies of noise power, E, and gain, F, behave much as in WN stimulation. Onset time of the impulse response, G, and the cut-off frequencies of the linear, H, and noise-free, I, coherences show similar evolution as seen with WN stimulation. The temporal pattern of the NS stimulus, J, displays long-term correlations (with no characteristic time constant), leading to a typical 1/*f* power spectrum trend and a probability distribution that completely departs from Gaussian (scale bars: 300 ms, 3 a.u. of light intensity).

**Figure 10 pone-0002173-g010:**
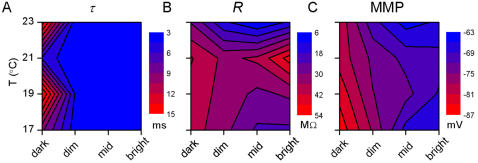
Membrane properties deduced from the current steps experiment. Voltage responses to the injected current steps were used to investigate the transmission properties of the photoreceptor membrane (Fig. S1). A, Membrane time-constant, τ, is greatly reduced from the dark-adapted state by dim light adaptation, but it reduces only slightly further with brightening BGs; i.e. the membrane ‘switches’ from a dark to a light-adapted state. B, Membrane resistance, *R*, displays a complex behavior in the light BG–temperature plane that correlates with the duration of the bumps, estimated from the noise power spectra (Fig. 6D). C, Mean membrane potential MMP shows that the light-induced depolarization increases by ∼15 mV from dark to bright BG, yet it is virtually temperature insensitive.

**Figure 11 pone-0002173-g011:**
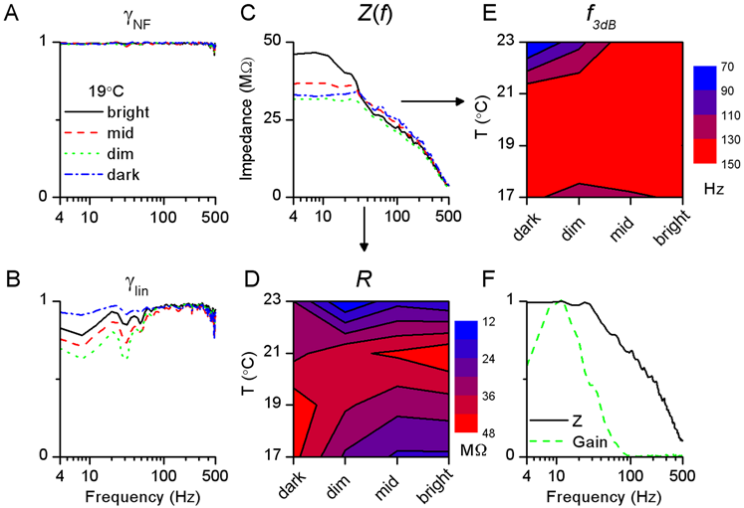
Dynamical properties of photoreceptor membrane investigated by WN currents. A, Noise-free coherence, γ*_NF_*, shows that the membrane can linearly translate WN current input into voltage output up to very high frequencies (500 Hz). B, Linear coherence, γ*_lin_*, shows only very small noise contamination over the whole frequency range. C, Impedance curves, *Z*(*f*), show that photoreceptor membrane acts as a low-pass filter. Data for A–C was recorded at 19°C in dark and at the three light BGs; the results at the other temperatures show nearly identical behavior. From the impedance functions we estimated the resistance, *R*, and the 3 dB cut-off frequency, *f*_3dB_. D, resistance estimate from WN stimulation strongly resembles the resistance estimated from the current steps experiment. E, Cut-off frequency is virtually constant and much higher than the cut-off frequency of the voltage responses to light. F, Plotting the normalized impedance (i.e. current-to-voltage gain) and the light-to-voltage gain clearly shows that, at the level of the photoreceptor soma, the membrane is not matched to filter out high-frequency phototransduction noise (shown for 19°C – mid BG, all conditions giving similar results).

**Figure 12:**
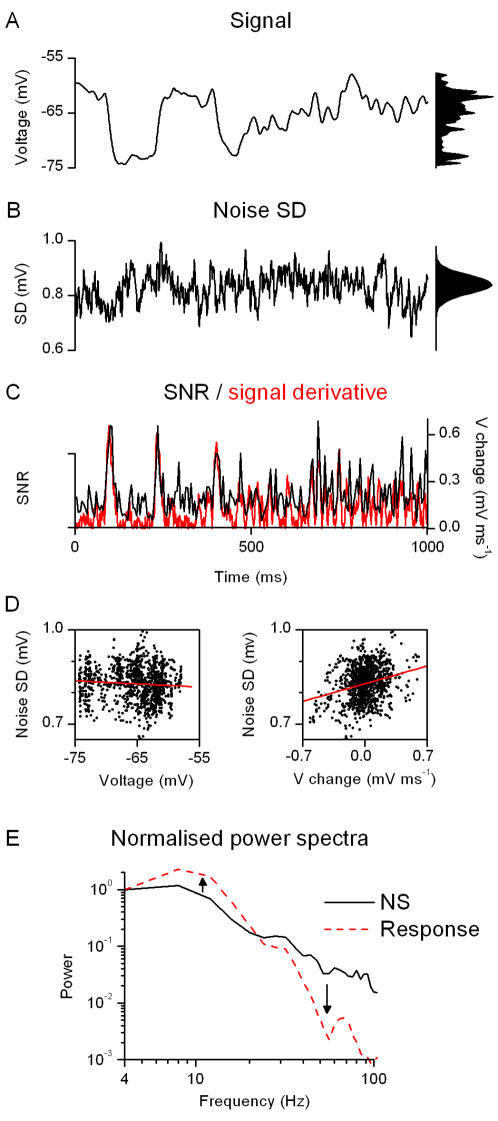
The photoreceptors enhance transient features of the stimulus and flatten the probability density of the transmitted frequencies. The reliability of temporal patterns in the photoreceptor responses is analyzed by comparing the average response, A (i.e. signal), to the time-dependent variability of the voltage responses, B (i.e. noise SD), evoked by a NS sequence. The probability distributions of these functions are shown in right. Noise SD is non-uniform across the stimulation pattern, calculated for every time-point across the voltage traces to the last 90 presentations of the NS light pattern (the first 10 showing an adapting trend), at the bright BG at 19°C. At every time-point (left) the spread of voltage values of the responses follows an individual distribution, varying from skewed to Gaussian; however, their overall probability distribution approximates a Gaussian (right). The changes in noise SD are then compared to the *SNR*, C, estimated by calculating the signal SD and the noise SD over 5 consecutive time points (using a 10-point window gives similar results). Notice that the amplitude of the rate of change in the signal, i.e. the absolute value of its time derivative (red trace), behaves similarly as the *SNR*, indicating that the locust photoreceptors encode most efficiently fast voltage changes. D, By ignoring their temporal order, 1000 values for (noise SD and signal) and (noise SD and rate of change of signal) are displayed as functions of voltage and rate of voltage change, respectively. The noise SD depends mostly on the rate of voltage change (linear fit slope = 0.08 ms, R = 0.26) and little on the instantaneous voltage value (linear fit slope = 0, R = −0.08). Notice that the noise SD does not only depend on the absolute value but also on the sign of the derivative. This could imply that there is an asymmetrical step in the phototransduction cascade, possibly arising from a process that involves 2 different time-constant for the transition between 2 different states (e.g. phosphorylated/non-phosphorylated). Such asymmetry would naturally occur if the 2 transitions involved 2 different enzymes. Alternatively, fast membrane dynamics or synaptic feedbacks could enhance depolarizing and hyperpolarizing response patterns asymmetrically. E, The normalized power spectra of the NS stimulus (ordinate units c^2^ Hz^−1^) and of one stretch of the photoreceptor response (as in Fig. 8, at bright BG, ordinate units mV^2^ Hz^−1^) illustrates how the cell enhances selected stimulus frequencies, whitening its output and increasing the entropy of transmitted signals.

**Table 1:** Q_10_ (17–27°C) for normalized data.

Q_10_ for:	dim BG	mid BG	bright BG
Dead-time^1^	2.0±0.7	1.8±0.4	1.9±0.5
	n = 8	n = 10	n = 9
Bump duration	1.9±0.7	1.6±0.6	2.5±1.0
	n = 3	n = 3	n = 3
Latency width	2.4±0.8	3.2±1.0	3.2±1.1
	n = 3	n = 3	n = 3
*K_1_* width	1.8±0.5	2.0±0.4	2.5±0.6
	n = 4	n = 6	n = 5
Gain τ^2^	1.7±0.5	1.7±0.5	2.2±0.7
	n = 7	n = 11	n = 9
Information WN^3^	2.7±1.6	2.3±1.0	2.6±0.9
	n = 5	n = 8	n = 8
Information NS^4^	1.6±0.6	2.0±0.8	2.0±0.7
	n = 4	n = 7	n = 6

Shown are mean±SD. Values were extrapolated from a smaller temperature range, using linear or exponential fits (details in Materials and Methods and Table S1). ^1^: As defined by the onset time of the impulse response, *K_1_*. ^2^: Characteristic time-constant defined as: τ = (*f*_3*dB*_)^−1^. ^3^: Information transfer rate (Shannon's formula). ^4^: Information transfer rate (triple extrapolation method).

### Recording procedures

The stimulus generation, data acquisition, and signal analysis was performed by a custom written program (BIOSYST, © M. Juusola, 1997–2008) based on the MATLAB programming language (Mathworks) using an interface package for National Instruments boards (MATDAQ, © H.P.C. Robinson, 1997–2008). More details on data acquisition and analysis are given in Juusola and Hardie [7] and Juusola and de Polavieja [6].

### Light stimulation

Light stimuli were provided with a green high-intensity light-emitting diode (Marl Optosource) driven by a custom-built LED driver. The light output of the LED was monitored continuously with a pin diode circuit. The LED light output was attenuated by neutral density filters (Kodak Wratten) to provide five illumination levels, or adapting backgrounds; each one log-unit apart, indicated as BG0 (10^7^ photoconversions s^−1^), BG-1, BG-2, BG-3, and BG-4. The light output range was calibrated by counting the number of single photon responses, bumps, [22] during prolonged dim illumination [31]. A Cardan arm system allowed free movements of the light source at a constant distance (85 mm) from the eye's surface with the light source subtending an angle of ∼2°, comparable to the reported values for the angular sensitivity of locust photoreceptors (from ∼1.2° when light-adapted to ∼2.6° when dark-adapted, [24]).

White-noise stimuli (WN) were generated using MATLAB functions. These pseudorandom contrast modulations had Gaussian amplitude distributions and were spectrally flat up to a chosen cut-off frequency (an example can be seen below, in Fig. 5J). WN stimuli with different cut-off frequencies were used in preliminary experiments (from 10 Hz to 10 kHz, at BG0 in the same cell), causing similar changes in the responsiveness and information transfer of the photoreceptors as reported earlier with blowfly photoreceptors [6]. We used 1s-long WN light stimuli and selected 200 Hz as the cut-off frequency, as this covered the range of frequencies locust photoreceptors could see (Fig. 1) without allocating much power on light patterns that are too fast for these cells to follow. 1 s-long naturalistic stimulus (NS) sequences were extracted from patterns downloaded from the van Hateren database [10]. They had a characteristic 1/*f*-type spectrum, a non-Gaussian distribution (an example can be seen below, in Fig. 9J), and were presented to the eye at 1 kHz. Four different NS patterns were first used to control that the results were independent of some peculiarities in the intensity variations but rather depended on the global statistics of the stimuli. The total power of the chosen NS pattern (the one that elicited the largest responses) and the total power of the WN stimulus pattern were very similar (differed by ∼4%). Therefore, any observed difference could be attributed to differences in the statistical properties of the stimuli.

Preliminary results (Figs. 1–3) indicated that three adapting BGs were representative of three different working regimes of the photoreceptor. These BGs are named as ‘dim’ (BG-3), ‘mid’ (BG-2), and ‘bright’ (BG0). Light contrast (*c*) was defined as a change in the light intensity (ΔY) divided by the mean light BG (Y_mean_):
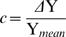
(1)For white-noise contrast modulation, ΔY was defined as the SD of the stimulus modulation. For naturalistic stimulation, the read-out values of the pin diode circuit monitoring the LED output were used without any calibration. These data are therefore presented using arbitrary units (a. u.) instead of contrast units. For direct comparison with WN, the NS data could be re-scaled in term of contrast units, but as its probability distribution departs completely from Gaussian, the SD of such a distribution has little significance. Alternatively, the NS values could be normalized by setting the lowest bound (when the diode is in effect off) to 0 and its highest (where the diode saturates) to 1. Either procedure left the results virtually unchanged. In the experiments, the cells were adapted to a selected light BG for >20 s before the WN or NS patterns were presented. Notice that because the WN patterns were superimposed on a constant light BG, whereas the NS patterns - that included longer dark periods - were not, the mean of the WN stimulus is higher than that of the NS stimulus, despite both having the same power.

### Current stimulation

To investigate how membrane properties of locust photoreceptors are modulated during light adaptation, we injected pulses or pseudorandomly modulated current into photoreceptors via the recording microelectrode. Electrode capacitance was carefully compensated before the current injection experiments. The use of a switched-clamp amplifier allowed us to record and monitor the true intracellular voltage and current during current injections and light stimulation [32].

### Data acquisition

Current and voltage responses were low-pass filtered at 1 kHz (KEMO VBF/23 low pass elliptic filter). These signals were sampled at 10 kHz for NS - 1 kHz was sufficient for WN signals as the corresponding light stimuli are cut-off at 200 Hz - then digitized with a 12 bit A/D converter (PCI-MIO-16E-4; National Instruments), and stored on the hard disk of a PC. The sampling was synchronized to the computer-generated stimuli and records of light and current stimulus, and voltage responses were stored during each recording cycle. To allow a fair comparison between WN and NS, the voltage response was re-sampled from 10 to 1 kHz. We checked that the results of the calculations were virtually independent on the sampling rate by re-sampling the data at 0.5, 1 and 2 kHz and repeating the analysis. The recording system, including the microelectrode, had a frequency response with a 3-dB high-frequency cut-off at 10 kHz or higher and therefore had negligible effect on the results. The noise level, estimated from measuring voltage fluctuations (SD) when the electrode was in the eye tissue, was <0.2 mV. Each experiment proceeded from the dimmest to the brightest adapting BG, at a given temperature.

### Data analysis

Most of the data analysis was conducted as explained in Juusola and Hardie [7] and in Juusola and de Polavieja [6]. Here we summarize the different stages of the analysis, using the example of a photoreceptor's voltage responses to light WN at 19°C. This allows us to define the relevant parameters used throughout this article and to highlight their biological significance, and by doing so to present the underlying assumptions and approximations of this study. We describe how the bump and membrane properties were investigated, and give a brief description of the triple extrapolation method used for estimating the information transfer rate of voltage responses to NS. We further define how the probability distributions were calculated to gauge the system's stationarity, and how Q_10_ values were estimated to quantify temperature-dependent changes.

#### Processing in the time domain: signal and noise analysis

Repeated presentations of the stimulus (WN or NS) evoked slightly different voltage responses. (WN data consisted of 31 responses, 101 responses were recorded for NS stimulation). In both cases, we rejected the first trace from the analysis as they systemically showed strong adaptive behavior. For each recording series, the averaged response is the ‘signal’, whereas the ‘noise’ is the difference between individual traces and the ‘signal’ (Fig. 1A). Hence for an experiment using *n* trials (with *n* = 30, WN, or *n* = 100, NS) there is one ‘signal’ trace and *n* ‘noise’ traces. In the simple case where linearity and additivity can be assumed (reasonable for WN, as discussed later on in the article), the noise term constitutes a random parameter that independently ‘contaminates’ each trial. In the case of NS, the noise term represents the probability distribution of all the possible response traces. The variance of the signal, σ*_S_* ^2^, and noise, σ*_N_* ^2^, were then calculated from the corresponding signal and noise traces. Additionally, we calculated the noise using the following method that prevents signal and noise from being correlated [33]. *n*-1 trials of an experiment consisting of *n* trials were used to compute the mean and the remaining one to compute the noise. This procedure is repeated for each possible set of *n*-1 responses, giving *n* uncorrelated noise traces. These two methods gave similar noise estimates.

We checked the distributions of signal and noise at the different experimental conditions. For the WN stimulation the distributions are very close to Gaussian at most conditions, although the noise distribution is skewed toward depolarization at very dim BGs, attributable to individual bumps, and the signal distribution is slightly skewed away from depolarization at the brightest BGs, attributable to saturation. When stimulating with NS, the distribution of the signal departs clearly from a Gaussian distribution, resembling the distribution of the response given below in Figure 11, whereas the distribution of the noise is Gaussian. However, in line with data obtained from photoreceptors of the flies *Calliphora* [6] and *Drosophila* [14] the variance of the responses differs at different moments of stimulation. This suggests that the ‘noise’ may play a role in the coding and the transfer of the visual information. Fourier analysis of signal and noise neglects the potential importance of ‘noise’, being an inherent limitation of this type of approach (see Discussion).

#### Calculation of the spectra

We calculated the corresponding power spectra for the mean stimulus, the signal, and every noise and response traces (Fig. 1B). They were divided into 50% overlapping stretches and windowed with a Blackman-Harris 4-term window [34]; then a fast Fourier transform algorithm was used to calculate their power spectra. Noise and response spectra were then averaged to improve these estimates (Bendat and Piersol, 1971). 〈|*C*(*f*)|^2^〉, 〈|*S*(*f*)|^2^〉, 〈|*N*(*f*)|^2^〉, and 〈|*r*(*f*)|^2^〉 are the stimulus (contrast, *C*), signal (*S*), noise (*N*), and response (*r*) power spectra, respectively, where || denotes the norm and 〈 〉 the average over the different stretches. From the spectra the different 3 dB-cut off frequencies, *f*_3 dB_, are calculated as the bandwidth at half height. Alternative ways of calculating the *f*_3 dB_, e.g. using the value where half the area under the curve is reached, gave virtually identical results. The coding properties are deducible from the *SNR*(*f*) (see below; Fig. 1C right), whereas the transfer properties can be derived from the cross spectrum between the stimulus and the signal (Fig. 1C left) as will be explained below.

#### The SNR spectrum: coding properties

For the WN stimulation, signal-to-noise ratio *SNR*(*f*) was calculated from the signal and noise power spectra, 〈|*S*(*f*)|^2^〉 and 〈|*N*(*f*)|^2^〉, respectively, as their ratio (Fig. 1C right). From 〈|*S*(*f*)|^2^〉, 〈|*N*(*f*)|^2^〉 and *SNR*(*f*) several parameters about the coding efficiency of the cell can be calculated (Fig. 2). Figures 2A and 2B show that brightening increases the signal power but reduces the noise power, respectively. In this article, when we mention power we mean the value integrated over the corresponding power spectrum. This result is further confirmed in the time domain from the independent measurements of the signal, σ*_S_* ^2^, and noise, σ*_N_* ^2^, variances, and their ratio, *SNR_t_*. We also estimated the information transfer rate from *SNR*(*f*) using the Shannon formula, which is applicable for this special case when both signal and noise distributions approximate a Gaussian [35]:
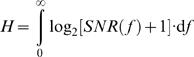
(2)where the lower limit of the integral was set to 2 Hz, because of the finite size of the recording (1 s), and the upper limit was set to 100 Hz, because of the unreliability of the signal at higher frequencies (Fig. 2C). Note that since 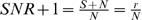, intuitively the information transfer rate measures the number of ‘coding states’ the cell uses. Two voltage states must be separated by at least *N* for being distinguishable and the useable voltage range *r* consists of *r*/*N* such states. The information transfer rate of the responses scales closely with the *SNR* over the tested luminances (not shown), vindicating the analysis.

From the *SNR*(*f*) we also calculated the linear coherence γ*_lin_* [33]:
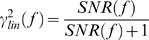
(3)Notice that 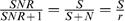; assuming that the system behaves linearly (see below for a test of this assumption), the more γ*_lin_* departs from unity the noisier the response at the given frequency (Fig. 2E).

#### The cross-spectrum of signal and stimulus: transfer properties

The cross-spectrum (Fig. 1C left) is calculated from the Fourier transforms of the signal and stimulus. It can be used for building several estimators that give insight about how a photoreceptor transforms the light stimulus into a voltage signal (cf. Fig. 3, below). Here we consider the cell as a filter; knowing its input (the controlled contrast stimulus) and output (the recorded voltage changes), we characterize its transfer properties. From the previous analysis it is clear that the noise is very small compared to the signal (*SNR_t_*∼70 at bright BG). In such practically noise-free (NF) conditions, we can use the noise-free coherence function, γ*_NF_*, to estimate the system's linearity [36]:
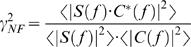
(4)where * denotes complex conjugate. γ*_NF_* is essentially the normalized signal and stimulus cross-spectrum. Assuming noise-free transmission, if γ*_NF_* is unity the system behaves linearly. This assumption is true in locust photoreceptors in most light conditions as seen in Figures 2E and 3G over a wide range of stimulus frequencies (see also [24]). This range, roughly between 4 and 60 Hz, is also where most information is carried (Fig. 2C). Therefore, for WN stimulation we can consider a photoreceptor as a linear filter and calculate its frequency response, *T*(*f*), as:
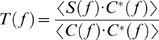
(5)
*T*(*f*) is a complex-valued function and therefore can be expressed in term of its norm *G*(*f*), the gain of the contrast-to-voltage transformation shown in Figure 3A, and its phase *P*(*f*), the phase-shift between the input and the output shown in Figure 3C, explicitly:
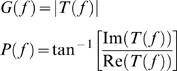
(6)


The contrast gain defines how a photoreceptor selectively amplifies the stimulus frequencies (Fig. 3A), which can be further characterized by its amplification integral and 3 dB cut-off frequency (Fig. 3B and D). It is easily seen from these figures that photoreceptor contrast gain increases with luminance but its frequency distribution remains relatively constant. Judged from their phase functions (Fig. 3C), photoreceptors are not minimum phase systems, but that their responses include a dead-time, as first described by [37]. To quantify this we first calculate the phase-shift of a minimum phase system, which would have the same gain:
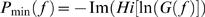
(7)where *Hi* is the Hilbert transform [38]; for details see Bracewell [39]. The phase-shift caused by the dead-time is then φ(*f*) = *P*(*f*)−*P*_min_(*f*). The dead-time was estimated over the flat frequency range (from 2 to 80 Hz) as ϕ(*f*)/2π*f* (Fig. 3C).

Another useful way to characterize the linear filter properties of a photoreceptor is to calculate its impulse response, or first-order Wiener kernel, *K_1_* (Fig. 3E), by taking the inverse Fourier transform of its frequency response:
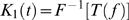
(8)This represents the impulse response. It summarizes in a graphically explicit way two important features of the frequency response: the amplification (estimated from the area of *K_1_*, not shown, that scales perfectly with the integrals of gain Fig. 3B), and the dead-time (estimated from *K_1_* onset delay, Fig. 3F, scales well with the dead-time estimated from the phase-shift, not shown). The time-to-peak values of the impulse responses are in good agreement with Howard [24].

#### Bump analysis: the elementary events and their distribution

We compared noise power spectra recorded in light and dark to gain insight to the elementary events, the bumps, which combine to form the total response. We first assume that the light-induced noise and the noise coming from other sources, i.e. intrinsic and instrumental, are independent and additive. Then, by subtracting the noise power estimated in darkness from the noise power at different light BGs - at a given temperature - we can estimate the light-induced noise power. We tested that the bump waveform follows a Γ-distribution [40] (this is shown in Fig. 6A, below):
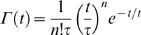
(9)We can obtain the two parameters *n* and τ by fitting a single Lorentzian, i.e. the Fourier transform of the Γ-distribution, to the experimental power spectrum:
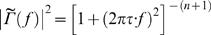
(10)where ∼ denotes the Fourier transform. From these two parameters the effective bump duration, i.e. the duration of a square pulse with the same power, is calculated:
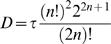
(11)As fitting the Lorentzian involves three free parameters: *n*, τ, and a scaling factor, the algorithm does not always converge satisfactory. To avoid biasing the results, in the fits *n* was fixed (*n* = 4, after seeing that it effectively retained the trend in the high-frequency tails of the power spectra). Although the values for τ differ with fixed *n*, the bump duration *D* remains remarkably close to its estimate when fitting with *n* as a free parameter.

Assuming that photon arrivals follow Poisson statistics it should be possible to extract the bump amplitude and rate from the light-induced depolarization of the membrane potential and its variance at different light BGs. However, as the membrane potential displays a complex, time-dependent behavior that not only involves depolarizing transduction currents but also hyperpolarizing activity of electrogenic ion-exchangers (see Discussion) such analysis is easily biased and therefore was not explored further in this article.

At this point we have estimates for the impulse response and the underlying events, bumps that construct it. The adapting bump model [40] assumes that a simple linear model can describe the summation of bumps. That is, the impulse response is the time-dependent product of arrival times of the bumps, i.e. latency distribution, and the bump waveforms. In other words, the impulse response is obtained by convolving the bump waveform by the latency distribution. Thus, the latency distribution can be inferred by deconvolving the bump waveform from the impulse response at each experimental condition. Since the impulse responses, particularly at dim conditions, can be noisy, we used log-normal fits [24], [41] of the impulse responses (Eq. 12) for the deconvolution to produce robust estimates.
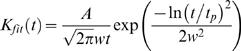
(12)where *A* is the amplitude, *w* the width, and *t_p_* the time to peak of the impulse response.

The adapting bump model can be explicitly written as:
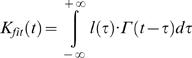
(13)where *l*(*t*) is the latency distribution we want to estimate and the lower limit of integral is taken to 0 – causality - and the upper limit to 80–120 ms, where the impulse response dies out. Impulse response and bump waveform were normalized before performing the deconvolution.

#### Current injection analysis: membrane properties

The properties of the photoreceptor membrane were investigated by injecting current steps. Hyperpolarizing steps lead to passive RC charging, characterized by a time-constant τ. This parameter was estimated by fitting a single exponential to the smallest hyperpolarization. We also constructed *V*-*I* curves, for which linear fits give the mean membrane potential, MMP (*V* value at *I* = 0), resistance, *R* (slope), and then capacitance, *C* (using τ = *RC*).

In locust photoreceptors potassium channels are activated at a voltage close to the resting potential, around −65 mV [25], [42]. We found a cell-to-cell variability in the resting potential of dark-adapted photoreceptors (see Discussion), which were often below −70 mV (∼−80 mV at 19°C for the photoreceptors presented in this article; Fig. S1). Around these voltages the potassium conductances were not activated, as it can be seen from the absence of rectification when hyperpolarizing (Fig. S1) that a truly passive RC charging occurs.

To further investigate the dynamic membrane properties of photoreceptors, we injected WN current [43] into the cells at different light and temperature conditions. The WN stimulus was 1 s-long, with a SD of 1 nA, and was presented 31 times (last 30 traces used in the analysis) at 1 kHz. Analysis of the voltage responses was conducted the same way as for the light stimulation. Here, the coherence (Eqs. 3 and 4) and impedance, *Z*(*f*), i.e. the gain of the frequency response (Eq. 6), are of a particular interest. The former characterizes the linear transfer properties of the membrane, and the latter its filtering with the same linear assumptions as previously. From the impedance we obtain two parameters: the DC component, the extrapolated value when *f* = 0 Hz, provides another independent estimate of the membrane resistance; its 3 dB cut-off frequency gives an index of low-pass filtering by the membrane.

#### Triple extrapolation method

When stimulated with naturalistic stimulation, NS, the distribution of the photoreceptor response was not Gaussian. In such conditions, the Shannon formula (Eq. 2) does not apply. We used the triple extrapolation method [6] to calculate the rate of information transmitted by the cell. Briefly, the voltage response is first digitized by dividing it into time intervals *T* that are subdivided into smaller intervals *t* = 1 ms. This digitization of the response can be understood as containing ‘words’ of length *T* with *T*/*t* ‘letters’. The mutual information between the voltage response *S* and the light contrast stimulus can be written as the difference between the total entropy:
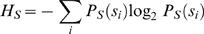
(14)and the noise entropy:
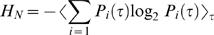
(15)where *P_i_*(τ)the probability of finding the *i*-th word at a time τ after the initiation of the trial. This probability *P_i_*(τ)is calculated across trials of identical NS. The values of the digitized entropies depend on the length of the ‘words’ *T*, the number of voltage levels ν and the *size* of the data file, *H^T^* ^,ν,*size*^. The rate of information transfer is then obtained taking the following three successive limits:
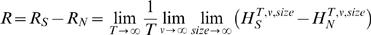
(16)We calculate these limits by extrapolating the values of the experimentally obtained entropies. Some practical considerations for the analysis are as follows. After removing the first trial (the first 1–20 traces when an adaptational trend could be seen; see the section about the system's stationarity in Discussion and Text S1 and Fig. S2), we typically used the next 100 traces. The voltage response was re-sampled from 10 kHz to 2 kHz, 1 kHz or 500 Hz (all giving similar values to the results shown here, using 500 Hz sampling rate) to remove high-frequency noise, and a response matrix of 1000/500 points×100 trials was obtained for the analysis. The order of the trials was also shuffled to minimize the effect of any remaining adaptational trend. The total entropy and noise entropy were then obtained from the response matrices using linear extrapolation with the following parameters: *size* = 5/10, 6/10,…,10/10 of data; ν = 8, 9,…,17 voltage levels; *T* ^−1^ = 3, 4,…,6 points. We also applied this analysis to WN data, providing values that closely scaled with the corresponding information transfer rate estimates (Eq. 2) [6].

#### Estimation of the probability distributions

To examine the stationarity of the system (see Text S1 and Fig. S2) joint probability distribution of the stimulus and the corresponding response were calculated for every (1 s-long) trace. To achieve this the parameter space, i.e. light intensity – voltage plane, with light intensity having arbitrary units between 0 and 1 and voltage running from −50 to −80 mV, is divided into 10×10 = 100 *cells* and we ‘follow’ the evolution of the system during each second. The amount of time it spends in a *cell* gives, after normalization, the associated probability. This 2D joint probability is then ‘projected’ onto the light and voltage axes to give the stimulus and response probability density distributions.

#### Quantifying the impact of temperature changes: calculation of Q_10_

Although the experimental data did not usually cover a 10°C temperature range, we could extrapolate reliable estimates for the Q_10_ of different parameters by fitting the data with a function corresponding to the observed trend. Table 1 displays the average and standard deviation (SD) of the different Q_10_ values, at each light BG. The temperature ranges used for evaluating the Q_10_ values, along with the details of their calculation, are given Table S1, as well as individual values obtained for each cell.

### Supporting Information

Supporting Information for this paper consists of two figures that vindicate the data analysis, five figures that highlight the repeatability and generality of the results, and one table that gives full details on the Q_10_ analysis. Fig. S1 shows the raw data of the current injection experiment that is used to measure the membrane properties (Fig. 10). Text S1 and Fig. S2 challenge the assumption that the photoreceptor voltage output is stationary by computing the joint probability distributions between the light intensity and voltage responses (see Materials and Methods). Fig. S3 shows the analysis of the voltage responses of another photoreceptor to WN light stimuli at different BGs and temperatures. Fig. S4 shows the analysis of the voltage responses of this photoreceptor to NS light patterns at different BGs and temperatures. Fig. S5 shows membrane properties of photoreceptors at different illumination and temperatures as pooled statistics of five photoreceptors, studied by current injection experiments. Fig. S6 shows analysis of the voltage responses of five other photoreceptors to WN light stimuli at different BGs and temperatures. Fig. S7 shows an analysis of the voltage responses of five photoreceptors to NS light stimuli (the same cells as in Fig. S6) at different BGs and temperatures. Together these data give further evidence to our finding that the overall response dynamics of photoreceptors, although not identical, show similar trends. Table S1 gives full details on the Q_10_ values for individual photoreceptors that are summarized in Table 1.

## Results

### 1. Light background and temperature are critical parameters for visual coding

Figure 4A shows 1-s long recordings of the membrane potential of a photoreceptor adapted to darkness and to three different mean illumination levels (light backgrounds, BGs) at 19°C, and the corresponding probability distributions. Light adaptation depolarizes the cell up to 10–15 mV, activating V-dependent channels [25] thus increasing the conductance and speeding up the transfer properties of the membrane (analysis of the membrane properties in section 4). Light adaptation also reduces voltage noise as seen by the narrowing distribution of the membrane potential. At bright BGs, photon shot-noise becomes negligible and the noise from the photoreceptor itself becomes very small, resulting in a probability distribution of the membrane potential close to the one seen at the dark-adapted state (Fig 4A, right). From these data, clearly one critical parameter in determining the photoreceptors' electrical properties is the light BG to which they are exposed.

Temperature is also an important factor governing the photoreceptor's responses to light. Figure 4B shows how warming a dark-adapted photoreceptor accelerates its voltage responses to a 10 ms-long light pulse of saturating intensity. By allowing the usage of higher frequencies, such accelerated responses should result in a more precise temporal coding when exposed to complex patterns, as will be shown below.

Hence for the locust, light background and temperature are not only ecologically relevant, as outlined in the Introduction, but they are also critical for its visual coding. Notice, however, that this work concerns only the encoding of the temporal intensity fluctuations from a single point in a scene and does not address the problem of image formation. While we should not forget this obvious limitation, the arguments about temporal coding are still valid. For a given spatial resolution (being limited by diffraction: [44] or by the optical properties of the rhabdom: [45]–[47]) an increased temporal resolution improves the visual acuity for moving objects [24], [48], [49].

### 2. Responses to WN: light background and temperature set the ‘encoding state’ of photoreceptors

#### 2.1. Coding and transfer properties

We recorded voltage responses to a WN light pattern at different adapting light BGs (dim, mid, and bright) and temperatures (17, 19, 21, and 23°C). The recordings we show here are from a single characteristic photoreceptor cell for the reasons mentioned above. Figure 5 shows the results for nine relevant parameters, as defined in Materials and Methods: signal power and corresponding cut-off frequency; noise power and cut-off frequency; information transfer rate; cut-off frequency of the gain function; onset time of the impulse response; cut-off frequency of the noise-free coherence; and cut-off frequency of the linear coherence. These parameters are displayed in the light BG-temperature plane as contour plots, together with the used stimulus.

Signal power increases both by warming and brightening (Fig. 5A), whereas noise power falls at brighter BGs, but is less influenced by the temperature (Fig. 5B). In general, the shape of the signal power at bright stimulation appears to mirror that reported from ocellar photoreceptors of locust at room temperature [50]. The increased signal power implies that the cell utilizes a larger voltage range for representing light patterns, whereas the decreased noise power establishes that the increased precision of voltage responses is attributable to brightening BGs. The combined effect is the increased information transfer rate (Fig. 5C).

The signal bandwidth used by the photoreceptor widens with warming, as seen in the increased cut-off frequency of the signal (Fig. 5D). The noise cut-off frequency (Fig. 5E) increases with brightening and warming, although showing a somewhat complicated behavior at bright BG (influenced by changing membrane properties; section 4). Thus, even though the total amount of noise in the voltage response is reduced at bright BGs, it contaminates the higher frequencies used by the photoreceptor. The cut-off frequency of the gain functions (Fig. 5F) behaves similarly to the signal cut-off frequency, indicating that the transformation from WN light to voltage response is mostly linear.

Warming accelerates voltage responses of photoreceptors (cf. Fig. 4B), allowing an effective encoding of higher stimulus frequencies (Fig. 5F, within 20–30 Hz; see also [18]). In accordance, Figure 5G shows how either brightening or warming reduces the delay (or onset) in the voltage responses, as seen in the linear approximation (impulse response, cf. Fig. 3E). The coherence functions (Figs. 5H and I) are also consistent with the observed dynamics. The bandwidth of the noise-free coherence (Fig. 5I) is always higher than the one of the linear coherence (Fig. 5H; in line with the findings at 19°C, Fig. 3H), which in turn is higher than the 3 dB cut-off frequency of the corresponding gain functions (Fig. 5F).

In general, locust photoreceptors appear to code efficiently WN light stimuli (gain) by reducing the noise (γ*_lin_*) at the frequencies where the signals are linear (γ*_NF_*). This finding supports the validity of the linear analysis for the given stimulation. The WN signaling may be non-linear to some extent, as seen in the deviation in coherence from unity when the stimulus spectrum departs from being flat (cf. Fig. 1B and Fig. 3G), but with such rapidly changing and linearizing [31], [37], [51] stimuli the frequencies where the photoreceptors transmit most information are linear (as reported for small sinusoidal contrasts: [24]).

#### 2.2. Bump noise analysis

By stimulating dark-adapted photoreceptors with a very dim BG (∼1 photoconversion s^−1^, BG-7), we were able to record single photon responses (Fig. 6A; or elementary responses) and directly test the assumption that their waveform (bump) follows a Γ-distribution. The bump parameters (amplitude = 3.2 mV, half-duration = 39 ms for the one shown in Fig. 6A) agree with the previous recordings [24]. Although the fitting algorithm did not converge satisfactory (the fitting was for ‘noisy’ individual bumps, not their averages), we could extrapolate ‘by hand’ the parameters that provided a good approximation for the recorded bump(s) (Fig. 6A), guided by the values obtained with the bump noise analysis (see below). Hence the bump waveform can be well approximated, as used in the following analysis.

We next compared noise spectra estimated in darkness and at different light BGs. This data allowed us to estimate the shape of the bumps summing up the light response (see Materials and Methods). Figure 6B shows the noise power spectra at different BGs and temperatures, whereas Figure 6C shows typical (normalized) bump waveforms estimated from these recordings for different BGs at 19°C and for different temperatures at the dim BG. From these bump waveforms we calculated the effective duration of the bumps (Eq. 11), displayed in the light BG–temperature plane (Fig. 6D). Essentially, neglecting the data point at the 21°C – at bright illumination (fully explained by considering the membrane properties, see section 4), brightening and warming accelerates the bumps. When *n* is fixed in the fitting algorithm (see Materials and Methods), the duration (*D*, proportional to τ) of the bumps describes entirely their shape, a scaling factor aside. This analysis implies that the elementary coding events are influenced both by light background and temperature. The adaptive state of the photoreceptor therefore affects all transduction reactions, not only on later stages, such as setting the overall gain.

The Q_10_ for the bump duration, *D*, was 1.9 at dim BG and 2.5 at bright BG (for 17–27°C; see Materials and Methods). These values suggest that the sources of the light-induced voltage noise have typical temperature-sensitivity of biochemical reactions, in line with the values reported for *Calliphora* [52], but they are somewhat larger than the values for *Drosophila* [8]. See Discussion and Table 1 for a fuller account of the Q_10_ values and their possible significance.

#### 2.3. Bump latency distribution

How do the elementary responses sum up to form the total response? Using the adapting bump model [40] we can estimate the bump latency distributions for the different light and temperature conditions (see Materials and Methods).

Figure 7A shows how the impulse responses are produced by the convolution of the bumps and their latency distributions. Figure 7B shows the latency distributions estimated under the same two conditions as in Figure 6C (19°C, dim BG) by deconvolving the bump waveform from the corresponding impulse response. Because the bumps are much briefer than the distribution of their occurrence, the latter sets the width of the impulse response (cf. the scales in Figs. 6C and 7B). Furthermore, as the bumps are minimum phase events [53], the dead-time, seen in the phase-shift and impulse response data (cf. Figs. 3F and 5G), comes from the latency distribution. Hence, bumps are delayed before they are actually produced on the photoreceptor membrane. The width of the estimated latency distributions in Figure 7C shows that brightening and warming enhances the synchronicity of bumps (see Discussion). Similar to findings from *Drosophila* [8], the Q_10_ of the latency distribution was larger than the Q_10_ of the bump duration. Here, however, the Q_10_ of the latency distribution also depends on the mean light BG, being the largest at bright illumination (Table 1). Thus, the light-adaptational acceleration of the voltage responses is inherently temperature-dependent: not only are the bumps themselves faster (cf. Fig. 6) but they also appear sooner and are better synchronized in warm conditions (Figs. 7B and 7C).

On a more general perspective, the phototransduction is modulated at different scales in space (microvilli, soma) and time (∼10 ms for producing a bump; ∼40 ms for summing up the bumps) to set the ‘encoding state’ of the photoreceptor, depending on the prevailing light and temperature conditions.

### 3. Responses to NS: input statistics are crucial for visual coding

Preliminary results shown in Materials and Methods (cf. Figs. 1, 2 and 3) with a WN stimulus suggested that locust photoreceptors are sensitive to the stimulus statistics, i.e. the stimulus and signal spectra show variable degrees of localized ripple caused by nonlinearities (cf. Figs. 1B and 3G). To investigate further how the stimulus statistics influence the coding strategy of the photoreceptor, we repeated the experiments using naturalistic light patterns, or NS, in lieu of WN at the same BGs and temperatures.

Figure 8A shows typical voltage responses to dim and bright NS at 19°C. At bright BG, the photoreceptor utilizes a larger voltage range to encode the light pattern, increasing the rate of information transfer with light BG (see below). When the averaged responses (signals) are normalized (Fig. 8C), we notice that the photoreceptor responds to the contrast stimulus with an increased delay at dim BGs but it can still follow the same transient changes. These dynamics imply that brightening NS would reduce delays in the voltage response without much affecting the photoreceptor's filtering properties. Thus, regardless of the prevailing conditions, locust photoreceptors accurately encode the naturalistic contrast input. This finding was confirmed by comparing the power spectra of the 3^rd^ and 30^th^ responses to the repeated NS at different light BGs (Fig. 8D). While the voltage range used for encoding the NS pattern increases for bright BGs at a given frequency, the range of frequencies (i.e. the bandwidth) effectively used by the cell remains virtually identical (the normalized power spectra overlap near-perfectly). Crucially, since this unexpected behavior is robust, seen in all photoreceptors (n = 8), we conclude that locust photoreceptors produce spectrally consistent neural representations of the naturalistic light patterns they encounter. We will show later that NS patterns are encoded into the rate of change of their voltage responses and consider the significance of this finding (cf. Fig. 12 and Discussion).

Long-term temporal correlations of NS result in a non-Gaussian distribution and a 1/*f* power spectrum (Fig. 9J). The selected NS and WN stimuli have the same total power. The voltage responses to NS showed that brightening and warming enhance the signal power, decrease the noise power, and increase the information transfer rate – as estimated by the triple extrapolation method - similar to the WN experiments (cf. Figs. 9A, B and C to [Fig pone-0002173-g006] [Fig pone-0002173-g007] [Fig pone-0002173-g008] Figs. 5A, B and C). The signal power, and thus the information transfer rate, is also affected by the ambient temperature, as in the WN experiments. In general, the information transfer rate of a photoreceptor (Fig. 9C) depends on the playback velocity of the selected pattern [6]; NS, played back at 1 kHz, gave slightly lower values than those of the estimated WN information transfer rate (Fig. 5C) -WN played back at 200 Hz (see Materials and Methods). Nevertheless, both of these values were similar to those estimated in locust ocellar photoreceptors using both Shannon's formula and the triple extrapolation methods [50].

The 3 dB cut-off frequency of the signal power spectra is remarkable constant over the different experimental conditions (Fig. 9D). This confirms the qualitative behavior observed in the raw data and signal power spectra (cf. Fig. 8B and D) and strikingly contrasts with the WN experiment (cf. Fig. 5D). The noise cut-off frequency (Fig. 9E) behaves similarly to the WN case, increasing with brightening and warming but also displaying a complex modulation at bright BGs. These findings indicate that the properties of the noise in the frequency domain are modulated by the same parameter for WN and NS, somewhat independently on the stimulation patterns. We will show in the next section that this parameter is the membrane resistance.

Although the frequency range used by the photoreceptor remains constant its frequency response broadens (Fig. 9F), indicating that the linear approximation may break down to some extent for the NS data. Comparing the actual response and the response, estimated by convolving the impulse response and the stimulus, validates this view: certain dynamic non-linearities along the processing stages deviate the voltage response by ∼10% from its linear approximation. Interestingly, this effect strongly varies along the response trace; in particular the linear approximation captures well the large transient changes. These findings together suggest that the encoding is mainly linear with a non-linear component dedicated to enhance ‘interesting’ patterns in the stimulus (see below).

The impulse responses describe well the reduced delays (Fig. 9G) observed in Figure 8B, supporting the idea that the encoding process is mainly linear. This finding is also supported by the behavior of the coherence functions, which behave very similarly to the WN experiment (cf. Figs. 9H and 9I to [Fig pone-0002173-g006] [Fig pone-0002173-g007] [Fig pone-0002173-g008] Figs. 5H and 5I). Thus, the non-linear amplification seems to be specific for certain patterns in the time domain and not simply specified in the frequency domain. Thereby, the transfer function of the photoreceptor would consist of a stationary, linear filter on which is added a non-linear filter that quickly adapts to the stimulus. The photoreceptors linearly enhance large transient features of the stimulus (see Discussion); whereas rapid non-linearities increase responsiveness to more subtle contrast changes. Of course, on a longer time scale slow non-linear processes are involved in realizing the light adaptation.

This break-down of the linear approximation may also partially reflect signaling constraints. In a very dim environment, a photoreceptor can respond to brightening but not to dimming inputs; thus, light contrasts are asymmetrically encoded. The situation is of course similar, but reversed, for saturation. By carefully choosing our light BGs and allowing the photoreceptors to fully adapt to the ambient light before the experiments, we prevented such saturation non-linearities (cf. the probability distributions for the voltage output Fig. 12A, below). However, many other types of constraints are also imposed upon phototransduction (numbers of microvilli and available molecules; the speed of bioreactions, refractory periods, energy supply-chains etc), each with particular dynamics. These complex constraints could contribute to some of the non-linearities in our data.

The main differences when stimulating the photoreceptors with NS, as compared to WN, are therefore that: (1) the photoreceptors make use of non-linear coding to enhance certain patterns in the stimulus, and (2) they produce outputs within a constant bandwidth. This may be evidence for a ‘preference’ (or ‘expectation’) of the photoreceptors for the long-term correlations (responsible for the 1/*f* spectrum) that occur in natural sceneries as is reported for spiking neurons of V1 area in the monkey cortex [54]; see Discussion.

### 4. Membrane properties participate in light adaptation

The voltage signal sent toward the first visual synapse is produced by charging the phototransduction (or light) current, and this process depends on the membrane properties [11]. To investigate how the transmission properties of the photoreceptor membrane change with light adaptation, we conducted current injection experiments.

#### 4.1. Membrane properties studied with current steps

Our aim here is to link the measured signaling dynamics (cf. Figs. 5 to [Fig pone-0002173-g006] [Fig pone-0002173-g007] [Fig pone-0002173-g008] 9) to the transmission properties of the photoreceptors membrane under similar experimental conditions. We recorded the voltage responses to depolarizing and hyperpolarizing current steps at different BGs and temperatures. From these recordings (cf. Fig. S1) we calculated the relevant transmission parameters of the photoreceptor membrane, shown in Figure 10.

The membrane behaves like a switch, changing transiently from a slow dark-adapted state to a fast light-adapted state. This transition is particularly clear for the membrane time constant, τ (Fig. 10A). There is a 4-fold reduction in τ from the dark-adapted state to dim conditions but its value is only slightly lessened when the adapting background is brightened further. Surprisingly, τ is hardly influenced by the ambient temperature.

The membrane resistance, *R*, has a more complex behavior in the light BG-temperature plane (Fig. 10B). It is difficult to assess what is responsible for these changes, but a comparison with Figure 5 shows what they cause. The duration of the bump waveform clearly correlates with *R* (cf. Figs. 6D and 10B). As the elementary events are very small, their speed is not limited by the charging of the whole membrane but, nevertheless, depends on the number of open channels. Hence, when the membrane time constant and resistance are proportional (assuming membrane capacitance remains constant, see below), bump duration and τ should correlate; the higher the number of open channels, the faster bumps. The correlation between the bump duration and the membrane resistance is further confirmed by normalizing these two parameters and comparing their behavior in the light BG–temperature plane: the similarity is >80% for 9 data points over 12 (3 light BGs×4 different temperatures), and >50% for the remaining 3.

The membrane capacitance, *C*, remains constant over a large area of the light BG-temperature plane (not shown), as expected for a photoreceptor surface area that stays about constant during the experiment. Deviations from this norm could result from difficulties in electrode compensation, as was also experienced during dynamic current injection (see below). The MMP depends only slightly on the ambient temperature, but it increases about 15 mV from dark to bright BG (Fig. 10C). This represents a dynamic balance between the light-induced depolarizing conductances, the hyperpolarizing voltage-sensitive conductances and the hyperpolarizing activity of ion-exchangers. All this activity reduces membrane resistance, and thus the membrane time constant, τ, consistent with the findings of Figures 10A–B.

To summarize: the transmission properties of the photoreceptor membrane are remarkably constant over the tested temperature range. However, the response accelerates when light depolarizes the photoreceptor. The membrane resistance has complex behavior, yet to be explained, that probably governs bump speed.

#### 4.2. Current WN stimulation and membrane dynamical properties

Using WN current injection it is possible to investigate the dynamic properties of the photoreceptor membrane, and describe them in the frequency domain [43]. The analysis is conducted in the same way as for the light stimulation (see Materials and Methods) with the most relevant parameters shown in Figure 11. Because noise is very small compared to signal, we first consider the noise-free coherence, γ*_NF_*. Figure 11A shows that the membrane translates the current input into voltage output linearly (∼99% unity) up to very high frequencies (>500 Hz). Similar to other preparations [7], [43], [55], rapidly changing current inputs (of different polarity) perturbate the voltage-dependent activation and relaxation dynamics of the photoreceptor membrane rather evenly, linearizing its voltage output [55]. The linear coherence curves, γ*_lin_* (Fig. 11B), show that the noise resides at relatively low frequencies (below 50 Hz). The membrane is noisier when light-adapted than when dark-adapted, but further brightening reduces the noise level, consistent with the narrowing voltage distributions (cf. Fig. 4A). The coherence functions are virtually temperature insensitive. The gain function of the corresponding frequency response between the current input and the voltage output is the complex impedance, *Z* [43]. Figure 11C shows how filtering properties (low-pass features of the impedance function) of the membrane change with light BGs at 19°C. The membrane resistance and cut-off frequency are calculated from the impedance functions for the different experimental conditions. The membrane resistance was estimated from the impedance function at 4 Hz, as this well approximated the DC values. These estimates (Fig. 11D) strongly correlate with the resistance measured in the current step experiment (cf. Fig. 10B), vindicating both analyses. The 3 dB cut-off frequency of the membrane impedance remains almost constant and high, ∼140 Hz (Fig. 11E).

The remaining differences may relate to the nature of WN current stimulus that leads to both depolarized and hyperpolarized potentials. During half of the time of the WN stimulation the membrane is exposed to depolarizing currents that activate voltage-sensitive potassium channels [25], [42], whereas hyperpolarizing current steps do not. Nevertheless, under all conditions the membrane cut-off frequency is at least 2 times higher (often more) than the corresponding cut-off frequency of the light-induced voltage responses. It is therefore unlikely that at the level of the photoreceptor soma the membrane would filter out high-frequency noise as it has been shown for other systems [56]. This behavior is graphically shown in Figure 11F where the gain of the light-induced voltage response and the membrane impedance are normalized and plotted together (shown at 19°C, mid BG; all tested conditions gave similar results).

Exploration of the dynamic properties of the photoreceptor membrane tells us that the apparent reduction of the time constant during light adaptation is useful for fast transmission of voltage responses but it does not play a clear role in noise reduction. The somatic membrane potential is able to follow current changes in a linear fashion at frequencies far beyond those produced by the phototransduction cascade in response to changing light inputs. In this respect, membrane conductance does not limit the speed of transmitted transduction signals. However, at the same time a complex modulation of the membrane resistance strongly correlates with bump speeds (see Discussion). Thus, it seems that the membrane helps determine the speed at which phototransduction currents elicit changes in membrane potential (the lower the resistance, the faster the bumps), but does not limit the speed of the underlying transduction reactions (the cut-off frequency is never reached). Hence, it is not the production of the bumps but their summation (the latency distribution) that limits the speed of the voltage responses (cf. Fig. 7).

## Discussion

The visual environment poses a serious encoding challenge to photoreceptors. Besides the vast, logarithmically scalable intensity range, the events of interest that occur within it come with a large range of velocities. In contrast, owing to many constraints in animal design, photoreceptors have evolved to rely on a small voltage range and limited transmission speeds to signal these events. Therefore, photoreceptors require complex nonlinear operations - jointly termed as light adaptation - to neurally represent the ever-changing visual world.

In this article, we investigated how light background and temperature modulate the size and speed of voltage responses in locust photoreceptors to random (WN) and naturalistic (NS, 1/*f*) light stimuli. We established that the response properties of these cells, as well as their adaptation properties, depend on the statistics of the stimulus; and showed how the phototransduction machinery and photoreceptor membrane are involved in the production of the voltage response. In the following we (1) recapitulate the main results, (2) propose possible explanations for the observed sensitivity to the stimulus statistics and discuss its significance, before closing on (3) the issue of intrinsic cell-to-cell variability.

### 1. Adaptation to ambient light and temperature conditions

We found that brightening or warming increases and accelerates voltage responses of photoreceptors (Figs. 5A, D, G and 9A, D, G). These dynamics are complemented by reduction in voltage noise (Figs. 5B and 9B) grading toward faster events (Figs. 5E and 9E). Because the frequency range of photoreceptors allocated for signaling contrast changes broadens (specifically seen with WN that contains proportionally more fast changing input patterns than NS; Figs. 5F, H, I and 9F, H, I), overpowering the noise (that tails off at only marginally higher stimulus frequencies, Fig. 6B), they can encode faster temporal events and transmit more information to the brain (Figs. 5C and 9C).

The increase in signaling speed (Figs. 5G and 9G) is caused by an *acceleration of* both the elementary phototransduction currents - leading to *bumps* (Fig. 6D) - *and their distribution* (Fig. 7C). We found that the bump waveform, or duration, is linked to the transmission properties of the photoreceptor membrane, as investigated by intracellular current injections (Figs. 10 and 11), showing a strong correlation between acceleration of the bumps and decrease in membrane resistance (cf. Fig. 6D to [Fig pone-0002173-g007] [Fig pone-0002173-g008] [Fig pone-0002173-g009] Figs. 10B and 11D). These findings provide new evidence for the hypothesis that significant adaptational changes in the speed and fidelity of responses occur at the level of light-gated ion channels [8], [57]. When more light-gated channels open in synchrony, the generated responses are larger and less noisy. Our results also highlight the combined action of light- and voltage-gated ion channels in enabling the photoreceptor membrane to perform a predominantly linear translation of phototransduction currents into the final voltage response (Figs. 3G, 5H, 9H and 11A) without limiting the throughput of these messages (Fig. 11F).

The latency distribution of bumps sets the ultimate speed limit for photoreceptor signaling and so determines the signal bandwidth. Following a light impulse, bumps scatter over a period that is much longer than the duration of an average bump (Fig. 7A). When considered together with the temperature-dependency of latency distribution (Table 1; Q_10_∼3), which is greater than that of the membrane-bound reactions responsible for the bump waveform (Table 1; Q_10_∼2), the findings suggest that the width of the latency distribution reflects, and is constrained by, enzymatic reactions at the early stages of the phototransduction cascade [7], [8]. Assuming that the phototransduction units are microvilli, and as such compartmentalized and separated [7], [58], [59], then keeping photoisomerized rhodopsins, which are few in number compared to other molecules in the phototransduction cascade (such as G-protein and phospholipase-C: [12]), active over prolonged but random periods should both improve the gain and integration of responses and reduce the noise from stochastic photon arrivals (seen as prolonged latency distribution in the experiments; Fig. 7). In this context, the adjustments of the latency distribution at different mean intensities and temperature that we see in locust photoreceptors (Figs. 7B and C; Table 1) may well participate in the general optimization strategy: minimizing the effects of photon noise and providing robust neural representations of the visual world at variable environmental conditions.

Finally, the study of the probability distributions at different times during the stimulation (Text S1 and Fig. S2) revealed that the system adapts very rapidly to the mean light level and so appears stationary, although prolonged adaptational trends could sometimes be seen. This behavior is probably a by-product of a photoreceptor regulating its ion homeostasis and, as such, may contribute only indirectly to the coding of temporal input patterns, for example at the synaptic level [5], [14]. In summary, the bump waveform is governed by the fast membrane, whereas the bump latency distribution reflects slower intracellular biochemical reactions; together their complex interactions (dynamic adaptation) enable efficient contrast coding at variable stimulus conditions.

### 2. Adaptation to stimulus statistics – temporal input patterns set the interactions between the fast membrane and slower intracellular reaction dynamics

Because the natural world consists of extended objects – and not of independent points - the visual images projected on the array of photoreceptors of exploratory animals are redundant [2], [16], dominated by slow intensity changes (low frequency or 1/f-type of correlations: [3], [9], [60]). Locust photoreceptors are sensitive to these global input statistics, as highlighted by their responses to naturalistic light patterns, having 1/*f* correlations. In the future, it would be possible to test whether the observed changes reflect long-term correlations in the stimulus. For example, one may design stimuli with different correlation schemes and compare the response properties when changing the time constant of the correlation term (if there is any), or modifying the strength of the correlations (changing γ in 1/*f* ^γ^-type correlations, e.g. [54]). Although using the same coding strategy as with WN, the impact of the light intensity and temperature is different for NS. During NS, signaling bandwidth of the photoreceptors did not increase with brightening or warming, in contrast to WN (cf. Figs. 9D and 5D, respectively; Figs. S3D and S4D; Figs. S6 and S7). Hence, the dynamic response and adaptation properties of photoreceptors appear to depend on the stimulus statistics used. How do we explain these findings, and what insight can we draw from them about phototransduction mechanisms?

Behavior of cells and their interrelationships are regulated by intracellular biochemical signaling [61]. This signaling has a high computational power, enabling complex functions [61]–[64]. Locust photoreceptors resemble other biochemical computational systems (e.g. *E. coli* flagellar motor; [65]) in one important way: their membrane dynamics are fast, whereas intracellular (phototransduction) reactions are slower (Fig. 11F). Coupling of the fast and slow dynamics can be responsible for the response to particular input patterns and their transient storage (dynamic adaptation) [7], [61]–[63], [66], [67]

The observed constancy of the signal bandwidth indicates that locust photoreceptors can represent naturalistic temporal input patterns (NS) with accurate rate changes in their voltage responses (Figs. 12A–D). To maximize the communication of important information (large or transient input patterns; i.e. to protect them against noise or being clipped by saturation: [6]), this is encoded into the rapidly rising or decaying responses, as these have the highest SNR (Fig. 12C; see also [14]). Indeed, it has been shown in *Calliphora* photoreceptors that only the early rising and decaying phases of voltage responses - evoked by unit-contrast pulses of different lengths - survive both the background noise and fast neural adaptation and therefore can accurately encode the actual contrast value of the stimuli [68]. These findings therefore indicate that the naturalistic contrasts encountered by locust photoreceptors continuously change the rise and decay rates of their responses, as regulated by dynamic interactions between the fast bump waveform and the slower bump latency distribution. This co-processing results in information being encoded into the speed (rate of change) of the voltage output. This system might have evolved to work with the naturally occurring 1/*f* statistics of light contrast, similar to most one-dimensional natural signals, e.g. [69].

There are at least three factors that are likely to contribute to the constant signal bandwidth to NS. Firstly, NS, unlike WN stimuli, have longer periods of relative darkness amongst brighter patterns. These moments appear to help to sensitize the phototransduction output so that sparse high-frequency contrast events can be amplified relative to the background. Because the same 1/*f* ratio of the light patterns is maintained at brightening backgrounds, the signal power spectrum, although now stronger, retains its characteristic low-passed shape across the range of illumination (Fig. 12E), providing the same 3 dB cut-off frequency. This whitening process, which works toward maximizing the entropy of transmitted signals, is equivalent to flattening the probability density of the transmitted frequencies [2], [3], [9], [70], [71]. Such rescaling of input, where fast contrasts (high stimulus frequencies) are enhanced at the expense of slower – redundant – background (slow stimulus frequencies), should necessarily make use of an adaptational (or ‘computational’) memory at the cellular level (e.g. the calcium concentration integrated over time: [72]) and other related nonlinearities (enhancement of signal transients by co-operative reactions: [73]; here at the level of ion-channels or by synaptic feedbacks, c.f. [14], [74]). Indeed, any system with coupled fast and slow dynamics will exhibit some form of ‘memory’ in its evolution. The sensitivity of photoreceptors to global, statistical features of the stimuli reported in this study serves therefore as an evidence for the existence of a ‘computational’ memory at the single cell level in a sensory system [7], [61]–[63], [66], [67].

Secondly, during naturalistic 1/*f* stimulation photoreceptors are on average *less depolarized by light* than during WN. This difference is because the mean of the WN stimulus is higher than the mean of NS stimulus for the same given light background. The more depolarized the cells the faster and more synchronized are their responses (Figs. 3 and 4A). Therefore, a naturalistic stimulus that switches between dark and light events (Fig. 8A) must on average generate a broader bump latency distribution than WN stimulus (cf. Fig. 7B), which carries more photons on the same unit time. Because the speed of the signals, and so too their bandwidth, is limited by the latency distribution of bumps (Fig. 7A), the signals stemming from broader bump latency distributions should be less influenced by the rapidly adapting bump waveforms than those at brighter WN stimulation.

Although these two factors together may explain the constant signal bandwidth from one light level to another, they fail to explain the lack of difference caused by warming, which reduces both bump duration and latency distribution (Figs. 6 and 7). To explain the constant signal bandwidth at different temperatures requires that the acceleration and deceleration of bump waveform and latency distribution are variable and scalable (or self-normalizing). The data clearly shows that Q_10_ of these and other critical parameters depends on the light BG, thus on the adapting state of the cell (Table 1), but gives no indication for the scalability - so that the total speed of the phototransduction reactions would not change as the speed differences of individual reactions would cancel out each other. Hence, this explanation seems unlikely. Instead, our findings are in line with an earlier suggestion [8] that the visual performance of poikilothermal insects follows environmental and behavioral constraints, promoting signal integration in cold and dark conditions and enhancing response speed when it is warm and bright. Thus, there must remain nonlinear processes involved in stabilizing the frequency range of voltage signals, which our analysis still cannot capture.

However, there is at least one more factor that can influence our interpretation of the data. It appears that to some extent the constant signal bandwidth could be attributed to limitations in the spectral analysis. When we average the responses to NS to eliminate the voltage noise (predominantly representing bump waveform) or calculate the spectral average from overlapping samples, these processes themselves may work toward stabilizing the estimated bandwidth (Fig. 9D). Therefore, our third point concerns about fallibility of the additivity assumption. Our results show that while the signal bandwidth (Fig. 9D) remains constant, the bandwidth of the frequency response (gain; Fig. 9F) increases with brightening and warming. This behavior is a clear indication that the simple signal and noise description is not fully appropriate here, as the average response (signal) lacks information about the stimulus that is actually encoded by the cell. We dissect this argument further.

One of the main assumptions of the signal and noise analysis is that noise is independent and additive. However, this may not be the case with insect photoreceptors (or possibly with any neuron). Although random, the ‘noise’ may depend upon the stimulus, or its history and so to be confused with adaptation. In fact, the noise distribution changes from one point to another in the voltage response. By calculating the noise distribution at each point, using a 10 ms window (Fig. 12B), we found that it remained always roughly Gaussian but with a rapidly changing SD, resembling findings from *Calliphora* [6] and *Drosophila* photoreceptors [14]. The noise SD did not strictly correlate with the absolute voltage value at the point where it was calculated, neither with the changes of the responses, i.e. the first derivative of the signal (Fig. 12D). Hence the system's memory of - or *dynamic adaptation* to - the preceding events may not only help the photoreceptors to produce invariable representations of the visual world, but also break the simple additivity assumption. Concurrently, our inability to separate adaptation from noise leads to an underestimated signaling performance of photoreceptors (Figs. 5C and 9C); see also [6].

We also found other differences in voltage responses of locust photoreceptors to WN and NS stimulation. Our data further reveals that, when comparing the signals in dim and bright NS (Fig. 8), the delay between transient changes in the stimulus and the corresponding response decreases with brightening. This reduced delay, or dead-time, can also be seen in the decrease of the *K_1_* onset time (Fig. 9F). Thus, the dead-time and the signal bandwidth of the photoreceptor are independent, hence are outcomes of different intracellular processes; see also [31]. This conclusion is also supported by the differing Q_10_s of the dead-time and latency distribution (Table 1) and the report that bump duration and latency are very weakly correlated. In locust this correlation can account for at most 7% of the global variance [24], similar to *Limulus* [75].

### 3. Cell-to-cell variability

We saw significant variability in the resting potential of locust photoreceptors (dark adapted, at 19°C) with values ranging from −60 to −80 mV. These cells all provided high quality recordings, so that the variability in their resting potentials did not reflect the quality of the microelectrode penetration or sealing. This could, of course, be caused by biased zeroing of the amplifier voltage before microelectrode penetration. However, this explanation seems unlikely as the extracellular potential, the reference value, remained stable and showed little noise, and so was easy to adjust throughout long-lasting experiments. The amplitudes of the voltage responses to a saturating light pulse also varied. This could partly reflect the uncontrolled positioning of the recording electrode within the cell. For instance, when the electrode was at a proximal position –close to the axon terminal- we could see a fast depolarizing transient similar to the ones reported to occur in fly photoreceptor axons [76] (arrow in Fig. 4B).

The cell-to-cell variability was unmistakable in the current injection experiments. For example, some photoreceptors displayed a significant voltage-dependent amplification when depolarized by current steps at certain light BGs, whereas others did this at different light BGs or, most frequently, failed to do so - such as the photoreceptor used in this article. Another example is the light-induced steady-state potential, or MMP, derived by fitting the *V*-*I* curves (at *I* = 0), that showed different behaviors, most commonly being more depolarized at brighter light levels (as shown in Fig. 10C) but sometimes the MMP would fall at brighter BGs. The evolution of the MMP seemed to critically depend upon the precise time-course of the experiment, i.e. the duration of the dark- or light-adaptation. The same experiment conducted in the same cell but at different times typically showed hysteresis. When the order and timing of experiments were fixed, the cell-to-cell variability was still unambiguously present in the recordings. The experiments were conducted at the same time of day, during the mid-afternoon, as a diurnal modulation of the membrane conductances in locust photoreceptors has been reported previously [25]. Indeed, exploring whether changes in the photoreceptor output occur in a circadian fashion [26], [27] would be an interesting avenue of research. The extreme stability of some of the recordings even suggests that such modulation could be studied within a single cell.

Judging all the evidence above, we argue that there is an intrinsic variability from one photoreceptor to another in the locust eye. The simplest explanation would be a differential expression of ion channels, possibly depending upon the position of the photoreceptor within the eye, which was not controlled in our experiments; see also [77]. Similar effect could be induced in a eye-location-specific manner by synaptic top-down regulation [14]. Different conductances, which would allow signals to be conducted at different speeds from different eye locations, would support the idea that the transmission properties of photoreceptors would correlate to the light statistics at different parts of the visual field. For example, the photoreceptors staring at the sky and the photoreceptors facing down will experience two very different optic flow fields as the animal moves [78], [79]. This hypothesis is testable: (1) by injecting dyes via the electrode at the end of the experiment (LY or neurobiotine) one can locate the photoreceptor within the eye; or (2) by measuring the zenith and azimuth of the light source one can map the receptive field of each cell in question. For constructing the functional organization of the average eye, one would then analyze a very large set of photoreceptors for each location.

## Supporting Information

Figure S1 Details on the current injection experiment. Voltage responses of the photoreceptor used throughout the main article (Figs. 4 to [Fig pone-0002173-g005] [Fig pone-0002173-g006] [Fig pone-0002173-g007] [Fig pone-0002173-g008] [Fig pone-0002173-g009] [Fig pone-0002173-g010] [Fig pone-0002173-g011] 12) to depolarizing and hyperpolarizing currents steps of different amplitudes were recorded when adapted to different BGs and temperatures. The stimulus, shown on the lower part of the figure, consisted of eight 150 ms currents pulses ranging from −0.5 to +0.5 nA, each presented 20 times, separated with 250 ms intervals. The average voltage responses are scaled by their mean DC components for each experimental condition. From these traces were calculated the parameters showed in Figure 10.
(6.71 MB TIF)

Figure S2 Joint probability distributions of the voltage responses to WN and NS light stimuli at 19°C. Joint probability distributions between the light intensity and voltage responses, and the individual probability distributions for the corresponding stimuli and responses are shown for the WN and NS stimuli, for the dim and bright BGs, at the 1st and the 30th s of stimulation, at 19°C, using the same cell used throughout the main article (Figs. 4 to [Fig pone-0002173-g005] [Fig pone-0002173-g006] [Fig pone-0002173-g007] [Fig pone-0002173-g008] [Fig pone-0002173-g009] [Fig pone-0002173-g010] [Fig pone-0002173-g011] 12). The response distributions at the 1st s are transposed on the corresponding distributions at the 30th s (dashed lines) to help to discern any adaptive trends. The results at the other temperatures were practically identical.
(9.73 MB TIF)

Figure S3 Repeatability and generality of our results: voltage responses to a light WN stimulus at different BGs and temperatures for 1 photoreceptor. Similar analysis as in Figure 5 in the main article, using another photoreceptor of exceptional stability. Note the temperature range investigated is slightly different from the one in Figure 5. A, Signal and B, noise powers. C, Information capacity. 3 dB cut-off frequencies of the signal, D, noise, E, and gain function, F. G, Dead-time in the voltage response, as estimated by the onset time of the impulse response. Bandwidths of the noise-free, H, and linear, I, coherences.
(6.04 MB TIF)

Figure S4 Repeatability and generality of our results: voltage responses to a light NS stimulus at different BGs and temperatures for 1 photoreceptor. Similar analysis as in Figure 9 in the main article, using the same photoreceptor as in Figure S3. A, Signal and B, noise powers. C, Information transfer rate, as estimated with the triple extrapolation method. 3 dB cut-off frequencies of the signal, D, noise, E, and gain function, F. G, Dead-time in the voltage response, as estimated by the onset time of the impulse response. Bandwidths of the noise-free, H, and linear, I, coherences.
(6.04 MB TIF)

Figure S5 Repeatability and generality of our results: membrane properties investigated by current injection experiments for 5 different photoreceptors. Voltage responses to injected current steps (A and B) or WN (C and D) were used to investigate how the membrane properties change with light BG and temperature. Data is pooled from 5 different cells as explained below. A, Membrane time-constant, tau, is greatly reduced from the dark- to the light-adapted state but is less affected by temperature (cf. Fig. 10A). Data at 20, 22 and 26°C are from 3 different cells. Warming increases the light-induced depolarization, B (cf. Fig. 10C). The amplitude of the depolarization and not the absolute value of the MMP is shown as the resting potential is variable from cell to cell. Data from the 2 cells, the first one recorded at 15 and 19°C, the second at 19 and 22°C, allowing to accurately rescale one relative to the other. C, Membrane resistance, R, displays a somewhat complex behavior in the light BG - temperature plane. D, The 3 dB cut-off frequency 3 dB, f3 dB, increases with both warming and brightening (cf. Fig. 11E) but is much higher than the cut-off frequency of the voltage response to light in any case.
(2.85 MB TIF)

Figure S6 Repeatability and generality of our results: voltage responses to a light WN stimulus at different BGs and 2 temperatures for 5 different photoreceptors. Similar analysis as in Fig. 5 and Fig. S3, using five photoreceptors of very good stability. A whole range of light BGs is investigated at 2 different temperatures for every cell. Note that the 2 temperatures used vary from cell to cell. Changes in the light BG - temperature plane is displayed for 9 parameters that help assessing changes in the coding and transfer properties of the photoreceptor. ITR stands for Information Transformation Rate, calculated using Shannon's formula.
(15.08 MB TIF)

Figure S7 Repeatability and generality of our results: voltage responses to a light NS stimulus at different BGs and 2 temperatures for 5 different photoreceptors. Similar analysis as in Figure 9 and Figure S4, using the same 5 photoreceptors as in Figure S6. ITR stands for Information Transformation Rate, calculated using the triple extrapolation method (see Materials and Methods). The signal bandwidth, shown here as the signal 3-dB cut-off frequency f3 dB, is remarkably constant, not only across the different temperature and light BG conditions for a given photoreceptor, but also across multiple photoreceptors, hence across multiple animals.
(15.08 MB TIF)

Text S1 (0.03 MB DOC)

Table S1 Details of the Q10 values for individual photoreceptors. Although the experimental data did not cover a 10°C temperature range in most cases, we could extrapolate reliable estimates for the Q10 of different parameters by fitting the data with a function corresponding to the observed trend. All the values were first normalized, i.e. the maximum value, usually at 17°C, was set to 1, then fitted with either a linear function (information transfer rate) or a first-order exponential decay (dead-time, bump duration, widths of the latency distribution and of the impulse response, gain, tau). The ratio of the value at 17°C over the extrapolated value at 27°C gives then the Q10 value. The characteristic time-constant for the gain was defined as the inverse of the corresponding 3 dB cut-off frequency. To accurately represent the timings of the latency distribution and of the impulse response we did not make any assumption concerning their shapes but calculated their areas when the maximum value (i.e. the value at the time-to-peak) was normalized to 1; this area is referred to as ‘width’. This analysis was conducted for several photoreceptors that were stable enough to repeat the experiments over a temperature range sufficient for reliable extrapolations. Table 1 displays the average and standard deviation (SD) of the different Q10 values, at each light BG. The values obtained for each cell along with the temperature ranges used are given in Table S1.
(0.04 MB DOC)
