# Supplementary figures and images for: Visual Coding in Locust Photoreceptors
Source: PLoS One. 2008 May 14;3(5):e2173. doi: 10.1371/journal.pone.0002173 (PMC2367440; doi:10.1371/journal.pone.0002173)

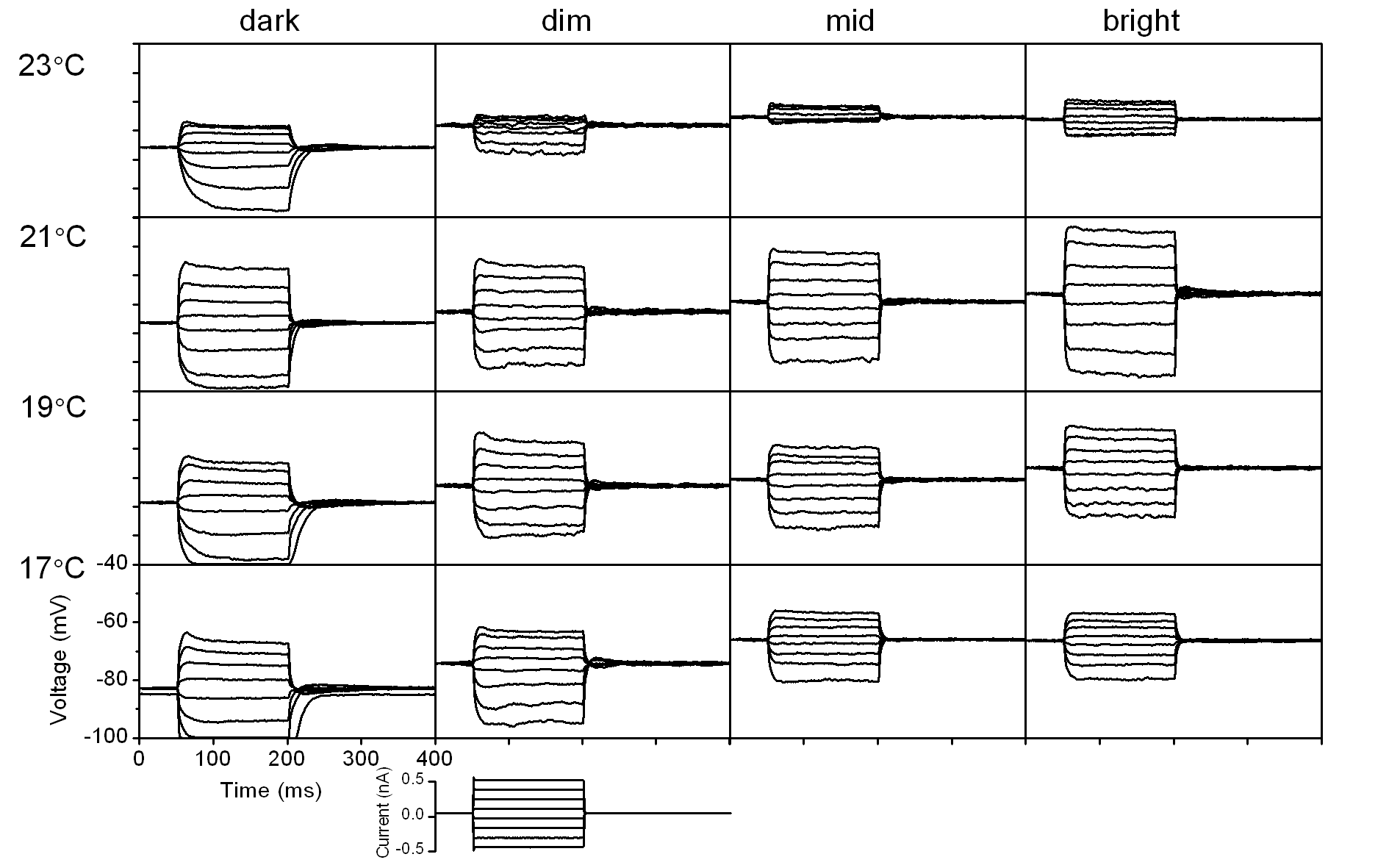

Supplement: Figure S1 — Details on the current injection experiment. Voltage responses of the photoreceptor used throughout the main article (Figs. 4 to 12) to depolarizing and hyperpolarizing currents steps of different amplitudes were recorded when adapted to different BGs and temperatures. The stimulus, shown on the lower part of the figure, consisted of eight 150 ms currents pulses ranging from −0.5 to +0.5 nA, each presented 20 times, separated with 250 ms intervals. The average voltage responses are scaled by their mean DC components for each experimental condition. From these traces were calculated the parameters showed in Figure 10. (6.71 MB TIF) [file pone.0002173.s001.tif]

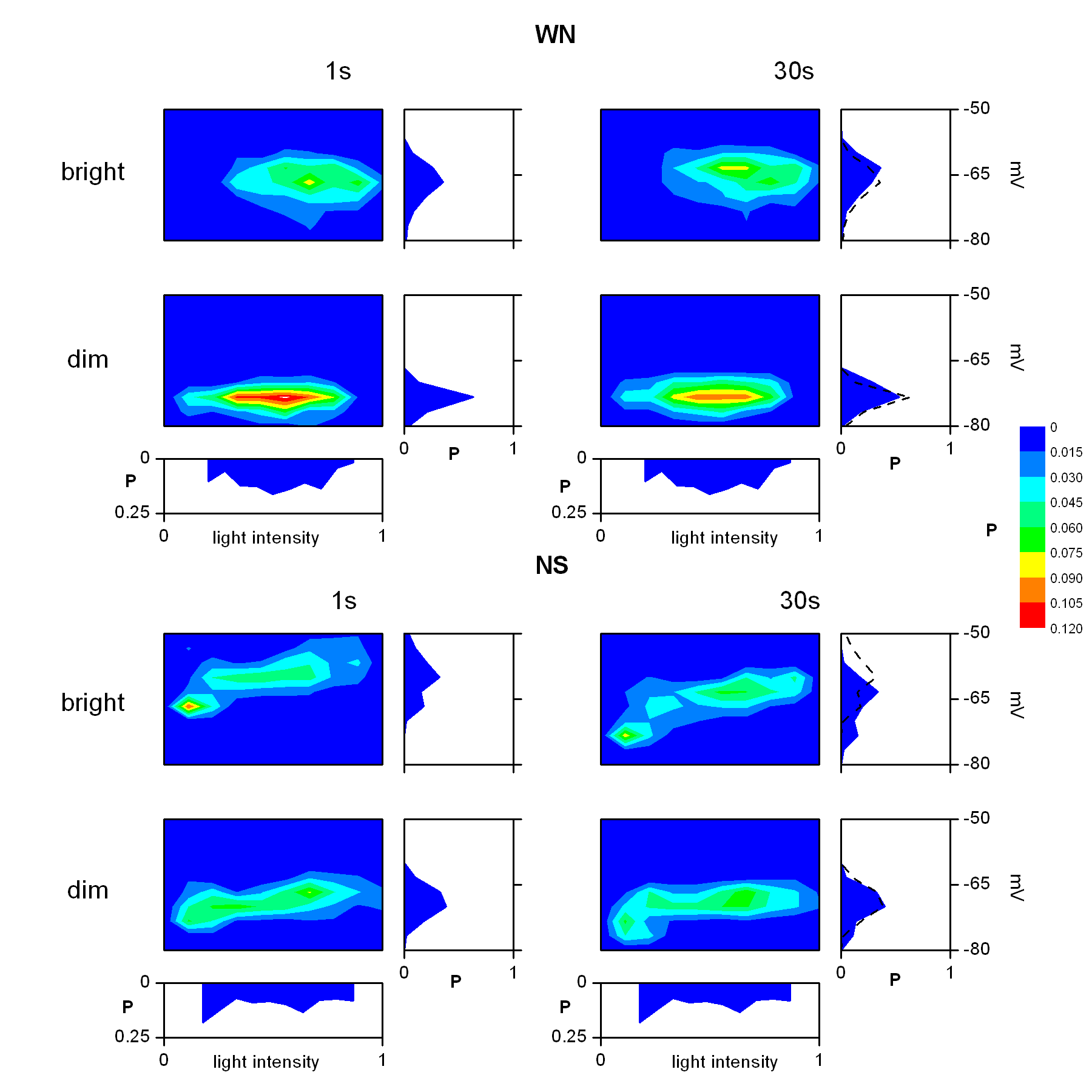

Supplement: Figure S2 — Joint probability distributions of the voltage responses to WN and NS light stimuli at 19°C. Joint probability distributions between the light intensity and voltage responses, and the individual probability distributions for the corresponding stimuli and responses are shown for the WN and NS stimuli, for the dim and bright BGs, at the 1st and the 30th s of stimulation, at 19°C, using the same cell used throughout the main article (Figs. 4 to 12). The response distributions at the 1st s are transposed on the corresponding distributions at the 30th s (dashed lines) to help to discern any adaptive trends. The results at the other temperatures were practically identical. (9.73 MB TIF) [file pone.0002173.s002.tif]

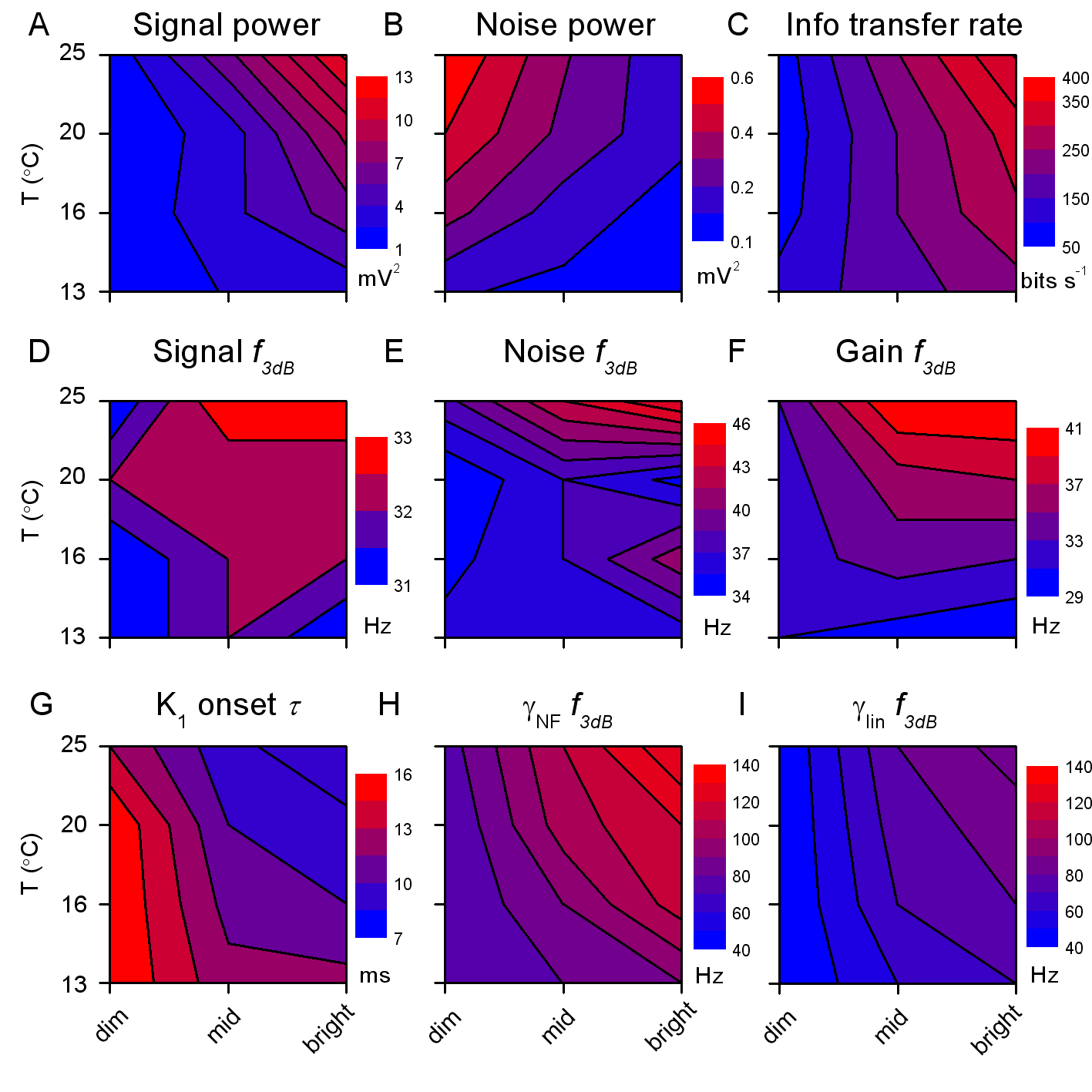

Supplement: Figure S3 — Repeatability and generality of our results: voltage responses to a light WN stimulus at different BGs and temperatures for 1 photoreceptor. Similar analysis as in Figure 5 in the main article, using another photoreceptor of exceptional stability. Note the temperature range investigated is slightly different from the one in Figure 5. A, Signal and B, noise powers. C, Information capacity. 3 dB cut-off frequencies of the signal, D, noise, E, and gain function, F. G, Dead-time in the voltage response, as estimated by the onset time of the impulse response. Bandwidths of the noise-free, H, and linear, I, coherences. (6.04 MB TIF) [file pone.0002173.s003.tif]

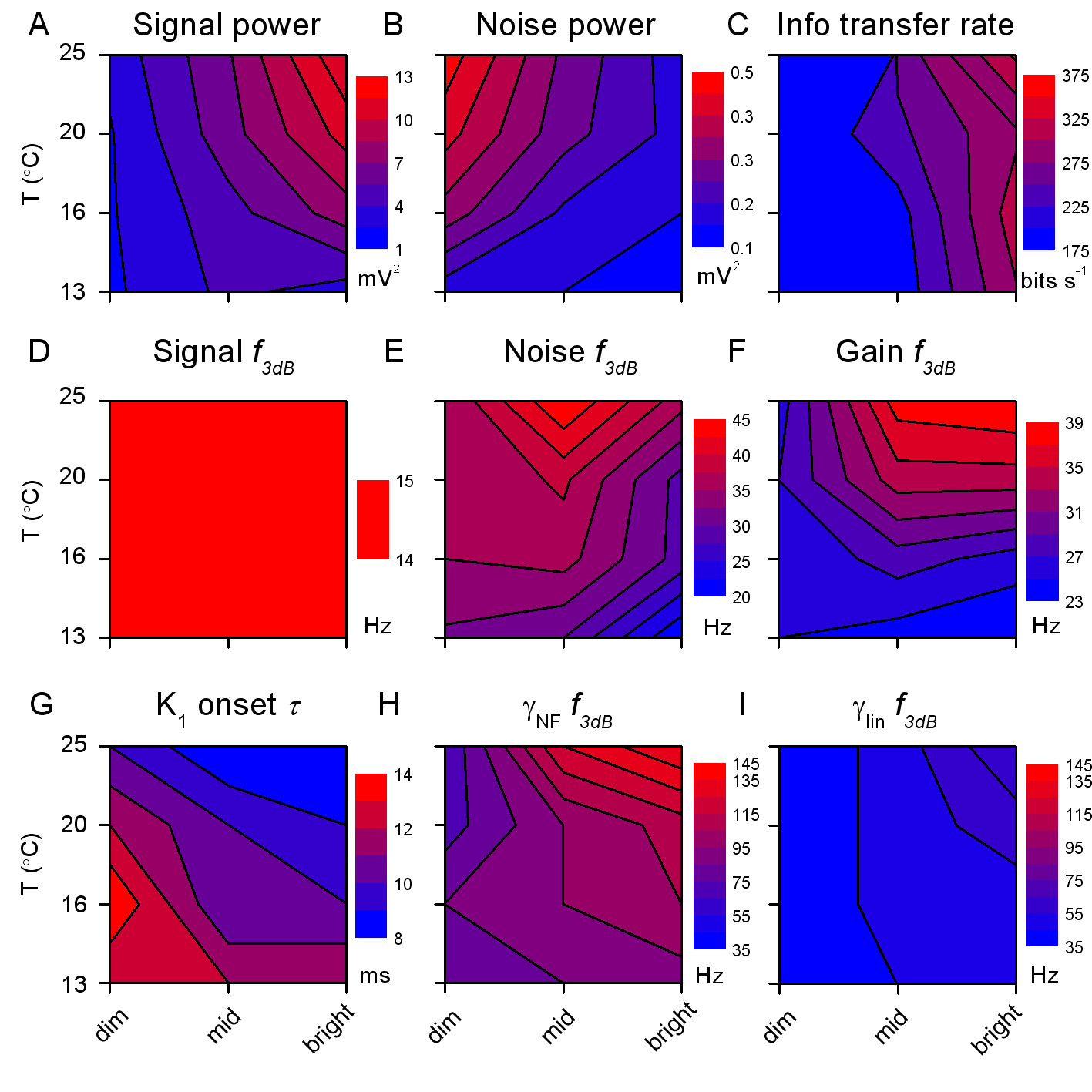

Supplement: Figure S4 — Repeatability and generality of our results: voltage responses to a light NS stimulus at different BGs and temperatures for 1 photoreceptor. Similar analysis as in Figure 9 in the main article, using the same photoreceptor as in Figure S3. A, Signal and B, noise powers. C, Information transfer rate, as estimated with the triple extrapolation method. 3 dB cut-off frequencies of the signal, D, noise, E, and gain function, F. G, Dead-time in the voltage response, as estimated by the onset time of the impulse response. Bandwidths of the noise-free, H, and linear, I, coherences. (6.04 MB TIF) [file pone.0002173.s004.tif]

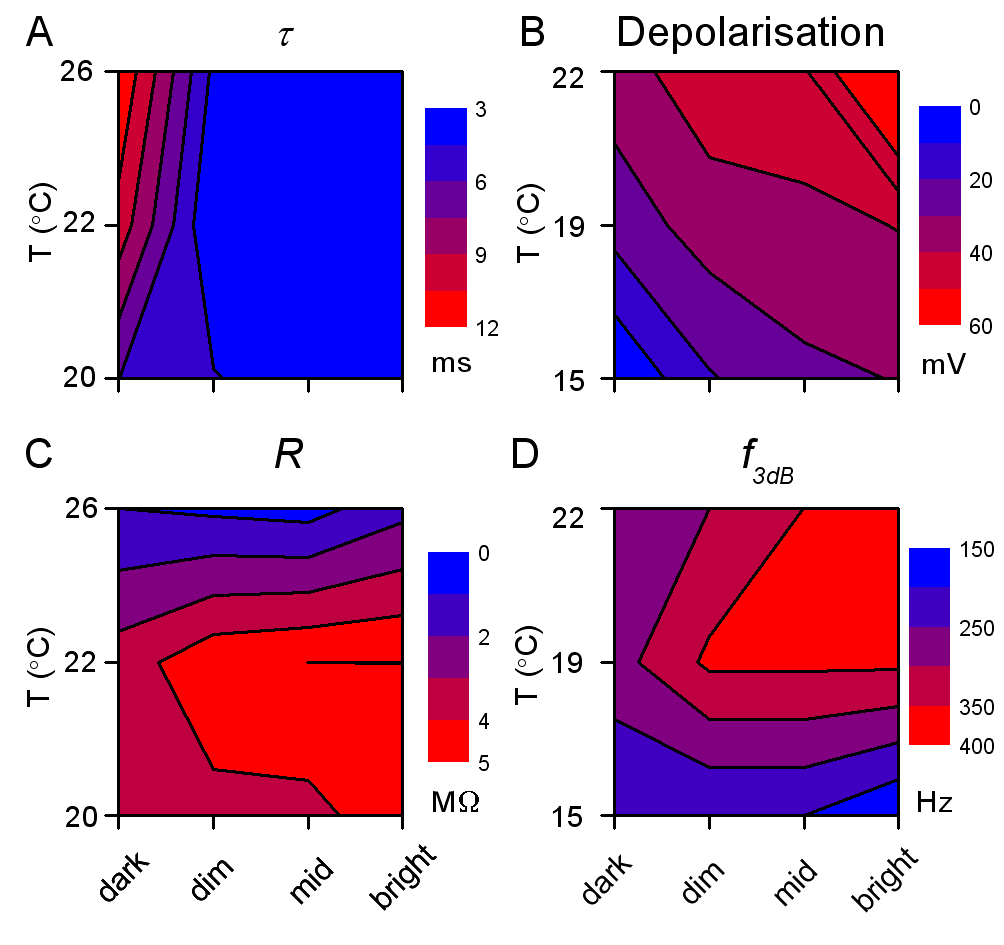

Supplement: Figure S5 — Repeatability and generality of our results: membrane properties investigated by current injection experiments for 5 different photoreceptors. Voltage responses to injected current steps (A and B) or WN (C and D) were used to investigate how the membrane properties change with light BG and temperature. Data is pooled from 5 different cells as explained below. A, Membrane time-constant, tau, is greatly reduced from the dark- to the light-adapted state but is less affected by temperature (cf. Fig. 10A). Data at 20, 22 and 26°C are from 3 different cells. Warming increases the light-induced depolarization, B (cf. Fig. 10C). The amplitude of the depolarization and not the absolute value of the MMP is shown as the resting potential is variable from cell to cell. Data from the 2 cells, the first one recorded at 15 and 19°C, the second at 19 and 22°C, allowing to accurately rescale one relative to the other. C, Membrane resistance, R, displays a somewhat complex behavior in the light BG - temperature plane. D, The 3 dB cut-off frequency 3 dB, f3 dB, increases with both warming and brightening (cf. Fig. 11E) but is much higher than the cut-off frequency of the voltage response to light in any case. (2.85 MB TIF) [file pone.0002173.s005.tif]

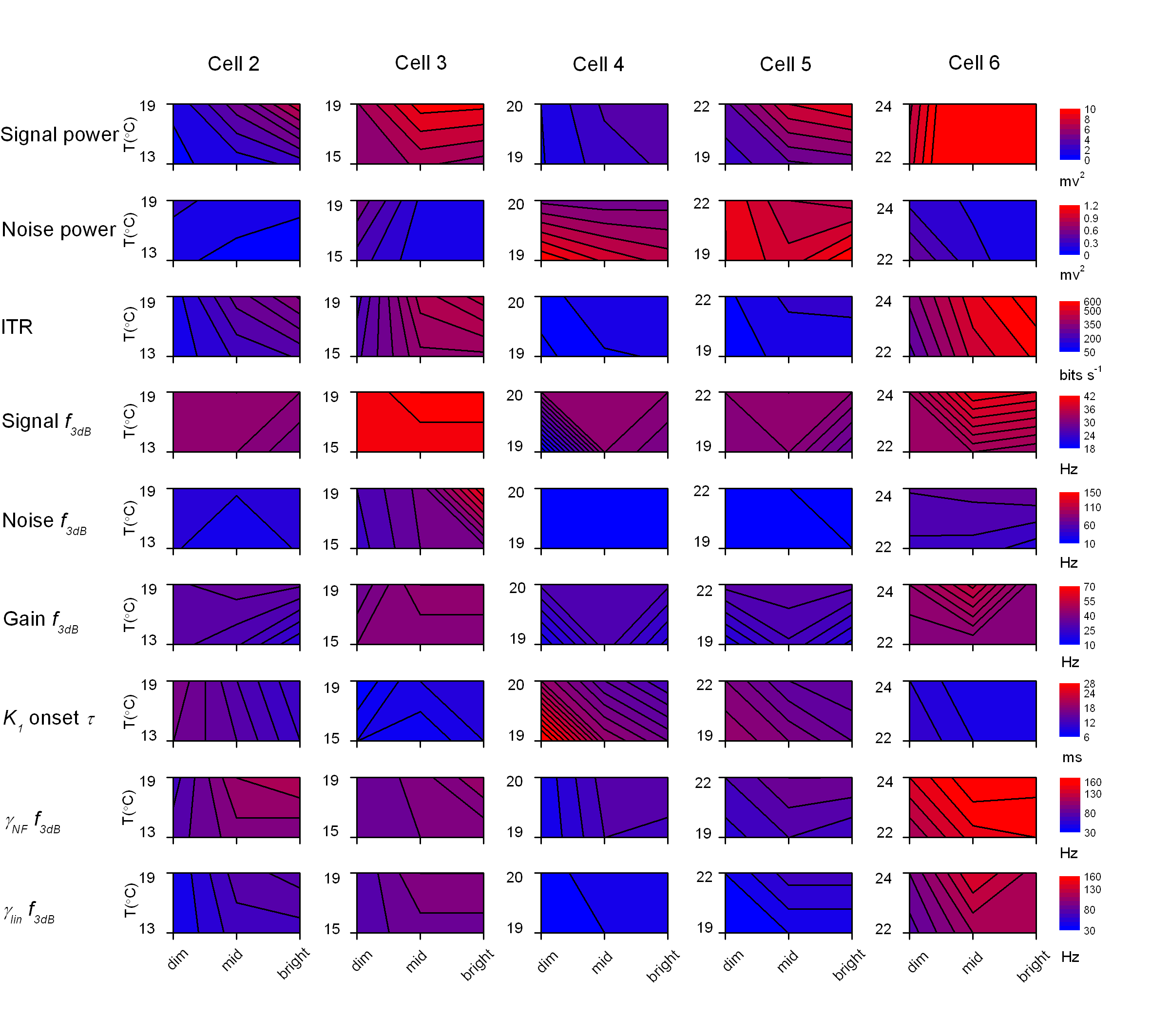

Supplement: Figure S6 — Repeatability and generality of our results: voltage responses to a light WN stimulus at different BGs and 2 temperatures for 5 different photoreceptors. Similar analysis as in Fig. 5 and Fig. S3, using five photoreceptors of very good stability. A whole range of light BGs is investigated at 2 different temperatures for every cell. Note that the 2 temperatures used vary from cell to cell. Changes in the light BG - temperature plane is displayed for 9 parameters that help assessing changes in the coding and transfer properties of the photoreceptor. ITR stands for Information Transformation Rate, calculated using Shannon's formula. (15.08 MB TIF) [file pone.0002173.s006.tif]

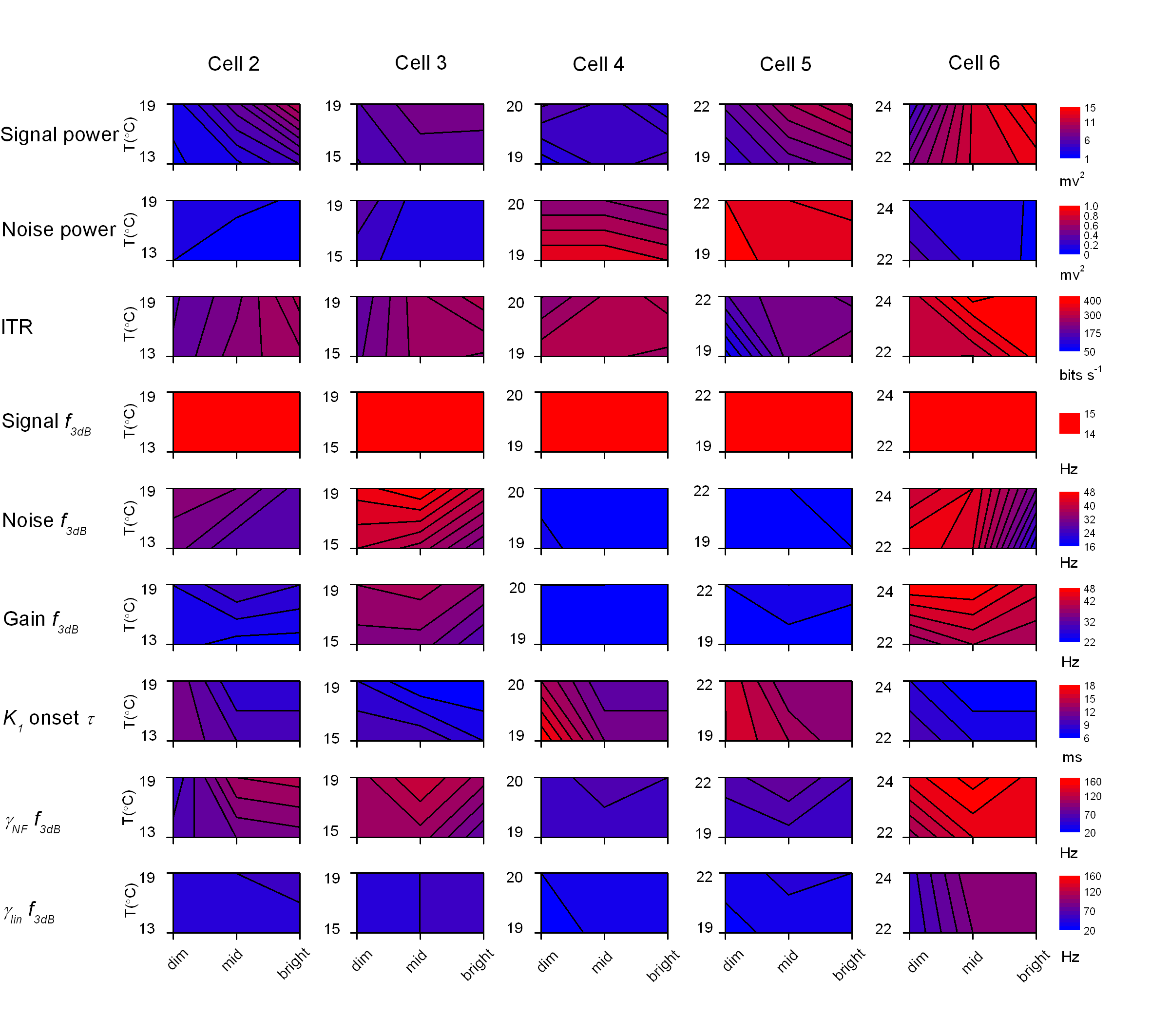

Supplement: Figure S7 — Repeatability and generality of our results: voltage responses to a light NS stimulus at different BGs and 2 temperatures for 5 different photoreceptors. Similar analysis as in Figure 9 and Figure S4, using the same 5 photoreceptors as in Figure S6. ITR stands for Information Transformation Rate, calculated using the triple extrapolation method (see Materials and Methods). The signal bandwidth, shown here as the signal 3-dB cut-off frequency f3 dB, is remarkably constant, not only across the different temperature and light BG conditions for a given photoreceptor, but also across multiple photoreceptors, hence across multiple animals. (15.08 MB TIF) [file pone.0002173.s007.tif]
