# Supplementary material for: Visual Coding in Locust Photoreceptors
Source: PLoS One. 2008 May 14;3(5):e2173. doi: 10.1371/journal.pone.0002173 (PMC2367440; doi:10.1371/journal.pone.0002173)
Supplement: Text S1 — (0.03 MB DOC) [file pone.0002173.s008.doc]

**Supplementary Text S1**

**Probing the system’s stationarity - the probability distribution approach**

*The voltage output of the photoreceptor is virtually stationary during the course of the experiment*

The signal and noise analysis relies on time-averaging over a string of voltage values and assumes that the studied system is stationary. To challenge this assumption we used an approach that does not rely on time-averaging. We calculated the joint probability distributions between the light intensity and voltage responses, and the individual probability distributions for the corresponding stimuli and responses for each repetition of the 1 s long trace of data (see Materials and Methods). Figure S2 shows these distributions for two light BGs (dim and bright) at two moments (traces for the 1^st^ and 30^th^ s; i.e. the 1^st^ and 30^th^ responses to the same 1 s long stimulus) for the WN and NS stimuli (shown at 19 ºC, no clear temperature effect was seen in this analysis). WN and NS produce very different distributions: WN distribution is practically symmetrical whereas NS distribution displays two ‘hot-spots’. These differences are attributable to the differences in the stimuli distributions.

For WN recordings, the brightening results in (1) a simple shift of the mean voltage (consistent with the current injection experiment) and (2) a spreading of the voltage distribution at bright BG (consistent with the increase of the information capacity). The probability distributions after 30s of stimulation are very close to that of the 1^st^ ones. In other words, the system is close to being stationary, showing only a small drift in the mean voltage.

For the NS recordings, the consequences of brightening are different from the WN case, apart from the shift of the mean membrane potential. In dim conditions the photoreceptor output was unable to clearly differentiate the ‘hot-spots’, the two voltage zones corresponding to the dark and light peaks in the stimulus distributions, seen at bright stimulation. When brightly illuminated the photoreceptor output switches frequently from one ‘hot-spot’ to another without spending much time between them. Here its voltage distribution resembles more the stimulus distribution than at the dim stimulation. Partially, this behavior should result from the lower SNR of dim stimulation, making it more difficult for the photoreceptor to reliably track fast changes in the stimulus pattern.

The shift of the probability distribution between the 1^st^ and the 30^th^ s of voltage responses (at bright stimulation) is caused by a global shift in the membrane potential during the first seconds of stimulation. Here the cell’s mean potential decays by ~ 6 mV following a single exponential with a time-constant of about 6 s, whereas the standard deviation of its responses changes by less than 5 % with no noticeable trend. Later, the adaptation trend dies out and we consider that the photoreceptor has reached a stationary state of responsiveness. Consequently, the probability distribution at the 100^th^ s is very close to the ones calculated at the 10^th^ s onwards, regardless of the stimulus intensity.

Both for WN and NS stimulation the 1^st^ trace was always removed from the analysis to minimize the impact of possible non-stationarities. We tested whether the findings were similar with or without removing any further adapting trends. From these tests the photoreceptor output showed reasonable stationarity during the recordings, so validating the way the data was analyzed throughout the article.

The second important finding is that the increase in the information transfer rate during bright NS stimulation is not only attributable to a broadening of the voltage range that the cell uses to encode the stimulus (as with WN stimulation) but also to the reduced delay in the voltage responses (see Fig. 8), which allowed the probability distribution to be ‘shaped’ toward the stimulus distribution. This is further evidence that photoreceptor coding is plastic, allowing the cell to be sensitive to general stimulus features under variable conditions.
