# Supplementary material for: Visual Coding in Locust Photoreceptors
Source: PLoS One. 2008 May 14;3(5):e2173. doi: 10.1371/journal.pone.0002173 (PMC2367440; doi:10.1371/journal.pone.0002173)
Supplement: Table S1 — Details of the Q10 values for individual photoreceptors. Although the experimental data did not cover a 10°C temperature range in most cases, we could extrapolate reliable estimates for the Q10 of different parameters by fitting the data with a function corresponding to the observed trend. All the values were first normalized, i.e. the maximum value, usually at 17°C, was set to 1, then fitted with either a linear function (information transfer rate) or a first-order exponential decay (dead-time, bump duration, widths of the latency distribution and of the impulse response, gain, tau). The ratio of the value at 17°C over the extrapolated value at 27°C gives then the Q10 value. The characteristic time-constant for the gain was defined as the inverse of the corresponding 3 dB cut-off frequency. To accurately represent the timings of the latency distribution and of the impulse response we did not make any assumption concerning their shapes but calculated their areas when the maximum value (i.e. the value at the time-to-peak) was normalized to 1; this area is referred to as ‘width’. This analysis was conducted for several photoreceptors that were stable enough to repeat the experiments over a temperature range sufficient for reliable extrapolations. Table 1 displays the average and standard deviation (SD) of the different Q10 values, at each light BG. The values obtained for each cell along with the temperature ranges used are given in Table S1. (0.04 MB DOC) [file pone.0002173.s009.doc]

**Supplementary Table S1. Details of the Q_10_ values for individual photoreceptors**

| Q_10_ for: | photoreceptor # | dim BG | mid BG | bright BG |
| --- | --- | --- | --- | --- |
| Dead-time^1^ | **1**  2  3  *4*  5  6  SC1  SC2  SC3  SC5  SC6 | **3.0**  2.6  -  *1.7*  -  1.6  1.2  -  2.6  1.5  1.5 | **2.1**  2.2  2.3  *1.5*  1.9  1.9  1.3  1.1  -  2.4  1.6 | **2.5**  2.3  2.3  *1.6*  2.2  -  1.6  1.2  -  1.8  1.2 |
| Bump duration^2^ | **1**  2  *4* | **2.3**  2.2  *1.1* | **1.3**  2.2  *1.2* | **1.7**  3.6  *2.1* |
| Latency width^3^ | **1**  2  *4* | **2.6**  3.1  *1.6* | **4.2**  3.2  *2.2* | **4.3**  3.3  *1.8* |
| *K_1_* width^4^ | **1**  2  3  *4*  5  6 | **2.0**  2.3  -  *1.3*  -  1.5 | **2.4**  2.3  1.4  *1.7*  1.9  2.2 | **2.8**  2.7  1.9  *1.8*  3.2  - |
| Gain *τ*^5^ | **1**  2  3  *4*  5  6  SC1  SC2  SC3  SC5  SC6 | **1.8**  1.9  -  *1.2*  -  1.7  -  1.1  -  2.6  1.7 | **1.9**  2.1  1.1  *1.4*  1.6  2.0  1.3  1.5  1.4  1.3  2.9 | **2.5**  3.3  1.6  *1.6*  1.9  -  1.6  3.0  1.4  2.7  - |
| Information WN^6^ | **1**  2  3  SC1  SC2  SC3  SC5  SC6 | **4.8**  3.8  -  -  1.1  -  1.4  2.6 | **3.9**  3.6  2.8  1.3  1.9  1.4  1.7  1.6 | **3.8**  3.1  2.8  1.5  2.2  1.9  3.5  1.6 |
| Information NS^7^ | **1**  2  3  SC1  SC2  SC3  SC5  SC6 | **1.7**  2.4  -  1.1  -  -  -  1.1 | **2.2**  2.6  2.5  1.1  -  1.1  1.2  3.0 | **2.4**  2.7  2.6  1.1  1.3  1.7  -  - |

From ^1^ to ^6^: data from WN stimulation. ^1^: onset time of *K_1_*, exponential fit. ^2^: calculated from eq. 10, exponential fit. ^3,4^: exponential fit. ^5^: characteristic time-constant defined as:$\tau=\left(f_{3dB}\right)^{-1}$, exponential fit. ^6^: information capacity, linear fit. ^7^: information transfer rate, linear fit.

The different temperatures used for the Q_10_ calculations are: for the photoreceptor #1 17, 19, 21, and 23 ºC; for the photoreceptor #2 17 and 21 ºC; for the photoreceptor #3 17, 19, 21, and 23 ºC; for the photoreceptor #4 20, 23, 25, 28, and 31 ºC; for the photoreceptor #5 17 and 19 ºC; and for the photoreceptor #6 20, 25 and 28 ºC. The photoreceptor #1 (values shown in bold in the table) is the one that is used throughout the main article (Figs. 4 to 12). For the photoreceptor #4 (values shown in italic in the table) is given the ‘raw’ Q_10_ (20-31 ºC), without any fitting. Photoreceptors used in the Supplemental Material: SC1 (Figs. S3 and S4) at 13, 16, 20, and 25 °C, Q_10_ (15-25 °C); SC2 (Figs. S6 and S7) at 13 and 19 °C, Q_10_ (13-23 °C); SC3 (Figs. S6 and S7) at 15 and 19 °C, Q_10_ (15-25 °C); SC5 (Figs. S6 and S7) at 19 and 22 °C, Q_10_ (19-29 °C); SC6 (Figs. S6 and S7) at 22 and 24°C, Q_10_ (14-24 °C).
